# The nature of halogen bonding: insights from interacting quantum atoms and source function studies

**DOI:** 10.1107/S2052252525000363

**Published:** 2025-01-27

**Authors:** Arianna Pisati, Alessandra Forni, Stefano Pieraccini, Maurizio Sironi

**Affiliations:** a Department of Pharmaceutical Sciences Università degli Studi di Milano via Mangiagalli 25 20133Milano Italy; b Department of Chemistry Università degli Studi di Milano via Golgi 19 20133Milano Italy; c CNR-SCITEC, Institute of Chemical Sciences and Technologies ‘Giulio Natta’ and INSTM RU, via Golgi 19, 20133Milano, Italy; Universidad de Oviedo, Spain

**Keywords:** interacting quantum atoms, IQA, quantum theory of atoms in molecules, QTAIM, source function, halogen bonding

## Abstract

A detailed study of the *X*⋯N (*X* = I, Br) halogen bonds in complexes formed by an extended set of substituted pyridines with *D*—*X* molecules (*D* = *X*, CN) is reported here. The nature of these interactions has been investigated at different (MP2 and DFT) levels of theory through Bader’s quantum theory of atoms in molecules (QTAIM) and Pendás’ interacting quantum atoms (IQA) scheme, focusing on the role of the local environment (*i.e.* the substituent on the pyridine ring and the halogenated residue) on the halogen bond features. We found that the exchange-correlation energy represents a substantial contribution to the IQA total energy, in some cases comparable to (I_2_ complexes) or even dominating (ICN complexes) the electrostatic term. Meaningful information is provided by the source function, indicating that the major contribution to the electron density at the bond critical point of the *X*⋯N interaction is derived from the halogen atom, while a much lower contribution comes from the nitro­gen atom, which acts as either source or sink for electron density. A relevant contribution from distal atoms, including the various electron-donor and electron-withdrawing substituents in different positions of the pyridine ring, is also determined, highlighting the non-local character of the electron density. The existence of possible relationships between binding energies, interaction energies according to IQA, and QTAIM descriptors such as delocalization indices and source function, has been inspected. In general, good correlations are only found when the local environment, external to the directly involved halogen and nitro­gen atoms, plays a minor role in the interaction.

## Abstract 2

Interacting quantum atoms and source function studies on a series of halogen-bonded complexes between substituted pyridines and *X*_2_ or *X*CN molecules (*X* = I, Br) focus on the combined role played by the *X* and N interacting pairs and their local environment.

## 1. Introduction

Non-covalent interactions (NCIs) represent a subject of ever-growing interest not only in view of the synthesis and characterization of new functional materials (Haque *et al.*, 2023 ▸; Molina *et al.*, 2017 ▸; Xiao & Fu, 2019 ▸; Bulfield & Huber, 2016 ▸; Moghadasnia *et al.*, 2024 ▸) as well as understanding biomolecular structure and function (Jena *et al.*, 2022 ▸; Xu *et al.*, 2014 ▸; Kojasoy & Tantillo, 2022 ▸; Walker *et al.*, 2023 ▸; Verteramo *et al.*, 2024 ▸), but also for the increasingly in-depth knowledge gained through theoretical and computational investigations (Brammer *et al.*, 2023 ▸; Kolář & Hobza, 2016 ▸; Wolters *et al.*, 2014 ▸; Phan Dang *et al.*, 2023 ▸; Scheiner *et al.*, 2020 ▸; Grabowski, 2021 ▸), where supramolecular systems often represent a test bench for assessing new methods and protocols (Jiménez-Grávalos *et al.*, 2021 ▸; Guerra *et al.*, 2024 ▸; Forni *et al.*, 2016 ▸, 2014 ▸, 2012 ▸). The fields of crystal and cocrystal engineering (Frontera & Bauzá, 2021 ▸; Nemec *et al.*, 2021 ▸; Saha *et al.*, 2018 ▸; Hajji *et al.*, 2023 ▸; Siddiqui *et al.*, 2024 ▸), eventually supplemented by experimental charge density studies (Forni *et al.*, 2019 ▸; Otte *et al.*, 2021 ▸; Eraković *et al.*, 2019 ▸; Shukla *et al.*, 2018 ▸; Thomas *et al.*, 2022 ▸), have greatly contributed to our knowledge and the potential of NCIs. Owing to the increasing body of structural and theoretical information, apparently well assessed definitions and concepts inherent to consolidated interactions, such as hydrogen bonds (Arunan *et al.*, 2011 ▸) and halogen bonds (XBs) (Desiraju *et al.*, 2013 ▸), need to be continuously upgraded and/or revisited (Grabowski, 2024 ▸; Scheiner, 2023 ▸; Varadwaj *et al.*, 2024 ▸).

Among NCIs, XBs represent, together with hydrogen bonds, the most widely studied interactions (Metrangolo & Resnati, 2012 ▸, 2008 ▸; Fourmigué, 2009 ▸; Politzer & Murray, 2013 ▸; Cavallo *et al.*, 2016 ▸), as testified by applications in different fields, spanning from biological systems (Parisini *et al.*, 2011 ▸; Wilcken *et al.*, 2013 ▸; Vargas Jentzsch & Matile, 2013 ▸; Auffinger *et al.*, 2004 ▸; Lu *et al.*, 2010 ▸; Cavallo *et al.*, 2016 ▸) to drug design (Jiang *et al.*, 2006 ▸; Bissantz *et al.*, 2010 ▸; Lu *et al.*, 2009 ▸), and the development of smart materials such as polymers (Lauher *et al.*, 2008 ▸), liquid crystals (Nguyen *et al.*, 2004 ▸), solid-state materials with peculiar electronic properties including superconductors (Kato *et al.*, 2002 ▸), and porous (Metrangolo *et al.*, 2009 ▸) and phospho­rescent organic materials (Bolton *et al.*, 2011 ▸).

An XB is the interaction between a covalently bonded halogen atom *X* and a nucleophilic acceptor *A*, according to the scheme *D*—*X*⋯*A*. The nucleophilic site is usually a lone pair on a heteroatom, such as oxygen (Kosmas, 2007 ▸; Kratzer *et al.*, 2015 ▸; Nelyubina *et al.*, 2011 ▸), nitro­gen (Hakkert & Erdélyi, 2015 ▸; Bartashevich *et al.*, 2014 ▸) or sulfur (Ford *et al.*, 2017 ▸; Eccles *et al.*, 2014 ▸; Hauchecorne *et al.*, 2011 ▸); an anion (Decato *et al.*, 2021 ▸; Fotović *et al.*, 2023 ▸; Mínguez Espallargas *et al.*, 2009 ▸, 2006 ▸); or a π-system such as a benzene ring (Luo *et al.*, 2022 ▸; Forni *et al.*, 2016 ▸, 2014 ▸, 2012 ▸). Moiety *D* may be both organic or inorganic in nature and diverse in size, ranging from another halogen atom (Schneider *et al.*, 2017 ▸; Alkorta *et al.*, 1998 ▸) to large biological residues (Borozan & Stojanović, 2013 ▸; Scholfield *et al.*, 2013 ▸).

The ability of a highly electronegative atom to interact with a nucleophilic site was first rationalized by Mulliken (1950 ▸). He interpreted an XB as deriving from the orbital interaction between the lone pair on *A* and the *D*—*X* antibonding orbital, thus assigning charge transfer (CT) character to the interaction, in addition to some degree of covalency. This model was subsequently confirmed by hybrid valence bond/molecular orbital (VB/MO) calculations, based on the block-localized wavefunction (BLW) (Mo *et al.*, 2007 ▸, 2011 ▸) method. BLW calculations on a series of XB complexes demonstrated that, except for weak interactions dominated by dispersion forces, CT is non-negligible or even the dominant contribution of most XBs (Wang *et al.*, 2014 ▸).

Much later, Politzer *et al.* (2010 ▸, 2007 ▸) explained that an XB can be attributed to the anisotropic electron density distribution around a covalently bonded halogen atom, whose valence electronic structure can be roughly described as *ns*^2^*np_x_*^2^*np_y_*^2^*np_z_*^1^, *z* being the direction of the bond. In agreement with such description, a concentration of electron density is found in the region orthogonal to the bond direction, and a depletion of electron density is present along the extension of the *D*—*X* bond. According to this model, the configuration of the bonded halogen explains the formation, in the electrostatic potential (ESP), of a positive region along the extension of the *D*—*X* bond, called a σ-hole, and a negative region around it (Politzer *et al.*, 2007 ▸, 2013 ▸; Murray *et al.*, 2009 ▸; Clark *et al.*, 2007 ▸; Clark, 2013 ▸; Kolář & Hobza, 2016 ▸). More recently, the existence of the σ-hole has been proven on a rigorous *ab initio* basis by means of spin-coupled (SC) VB calculations, and associated with a contraction of the SC orbitals describing the *p_z_* lone pair, while the negative belt around the halogen atom, observed only when bonded to electron-withdrawing groups of medium strength, is to be ascribed to a reduced contraction of the SC orbitals corresponding to the *p_x_* and *p_y_* lone pairs (Forni *et al.*, 2024 ▸; Franchini *et al.*, 2020 ▸). The features of the σ-hole depend on both the nature of the halogen atom [*i.e.* its polarizability and electronegativity (Messina *et al.*, 1998 ▸; Metrangolo *et al.*, 2002 ▸; Politzer *et al.*, 2010 ▸; Clark *et al.*, 2007 ▸)] and the chemical environment, in particular the electron-withdrawing ability of *D* and the charge-donor propensity of *A* (Lo *et al.*, 2012 ▸; Riley *et al.*, 2011 ▸; Kolář *et al.*, 2014 ▸). The strength of XB interaction has been proven to correlate with the ESP value on the σ-hole (Politzer *et al.*, 2007 ▸; Murray *et al.*, 2009 ▸; Riley *et al.*, 2009 ▸, 2011 ▸). According to SCVB calculations, different descriptors associated with the interaction, such as the overlap between the involved SC orbitals, their shapes and the Chirgwin–Coulson weights of the SC structures point to a VB picture of halogen bonding as due to a shift/delocalization of the Lewis base lone pair towards the halogen atom (Franchini *et al.*, 2019 ▸; Forni *et al.*, 2024 ▸). Moreover, it was reported that one of the electrons of the Lewis base lone pair is localized on the halogen atom in the direction pointing towards the *D* group, highlighting the importance of the CT contribution to the interaction, in agreement with the Mulliken model and BLW calculations (Wang *et al.*, 2014 ▸).

Most computational studies on halogen bonding, and in general on NCIs, are focused on global quantities, such as interaction energies and their decompositions, dipole moments, CT and so on, while very little is known about atomic contributions, in particular those coming from the donor and acceptor atoms directly involved, to determine these quantities. Such knowledge would provide important insights into the tunability of NCIs.

In this context, invaluable information can be acquired through the quantum theory of atoms in molecules (QTAIM) (Bader, 1991 ▸, 1990 ▸; Popelier *et al.*, 2000 ▸; Popelier, 2000 ▸) and the related interacting quantum atoms (IQA) approaches (Blanco *et al.*, 2005 ▸; Francisco *et al.*, 2006 ▸; Guevara-Vela *et al.*, 2020 ▸), which provide a partitioning scheme for the charge distribution of a system (and related properties) and its total energy, respectively, allowing us to gain insights into the specific role of interacting atoms in the formation of an XB (Alkorta *et al.*, 2020 ▸). Within QTAIM, a powerful tool is provided by the source function (SF) concept (Bader, 1990 ▸; Bader & Gatti, 1998 ▸; Gatti, Saleh & Lo Presti, 2016 ▸; Tantardini *et al.*, 2016 ▸), describing any local value of a scalar function (*e.g.* the electron density) as due to the contributions from all other points of the molecular space, therefore providing valuable chemical insights into covalent and non-covalent bonding.

In this work, we report the results of an investigation on the *X*⋯N XB performed through QTAIM, SF and IQA approaches. Inspired by the study of Bartashevich *et al.* (2014 ▸) on the I⋯N XB in substituted pyridines, we considered, for XB acceptors, the same set of pyridines, while for XB donors we examined, aside from the previously investigated I_2_ case, Br_2_, ICN and BrCN molecules, to evaluate the effect of the nature of both the halogen and the attached substituent on the XB interaction. Several relationships between binding energies, interaction energies (according to the IQA scheme) and QTAIM descriptors have been examined, based on different (MP2 and DFT) levels of theory.

## 2. Methods

According to QTAIM, atoms are viewed as disjointed and exhaustive regions of space, or basins (Ω), bound by zero-flux surfaces of the electron density gradient. The interatomic surfaces, for each point **r** on the surface *S*(**r**), follow the relation
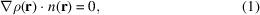
∇ρ(**r**) being the gradient of the electron density, ρ(**r**); and *n*(**r**) being the unitary vector normal to the surface at point **r**. The points where the first derivatives of ρ(**r**) vanish are defined as critical points, and the lines of maximum electron density connecting two critical points are known as bond paths.

The electron-pair sharing between two basins *Ω_A_* and *Ω_B_* connected by a bond path can be described in terms of the delocalization index (Bader & Stephens, 1975 ▸; Fradera *et al.*, 1999 ▸; Bader *et al.*, 1996 ▸):
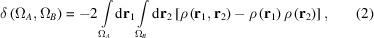
where ρ(**r**_1_, **r**_2_) is the pair density (*i.e.* the probability density of finding a pair of electrons at the volume elements d**r**_1_ and d**r**_2_); and 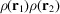 is the uncorrelated component of the pair density, which provides the probability of concurrently finding two independent electrons in positions **r**_1_ and **r**_2_. The delocalization index is therefore associated with the magnitude of the exchange of the electrons in the basin of atom *A* with those in the basin of atom *B*. This has been demonstrated to reflect the bond order (Outeiral *et al.*, 2018 ▸).

A useful tool to quantify how distant atomic interactions affect the delocalization between the two interacting basins is the SF (Bader, 1990 ▸; Bader & Essén, 1984 ▸; Bader & Gatti, 1998 ▸; Gatti, Saleh & Lo Presti, 2016 ▸; Tantardini *et al.*, 2016 ▸). This function is derived from the consideration that the total electron density at any reference point **r** can be seen as determined by local contributions from each point **r**′ in space, according to
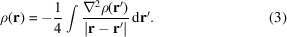
Partitioning the whole space into atomic basins allows us to replace the integration in equation (3) with a sum of integrations over atomic basins Ω*_i_*, each of them providing the contribution SF(**r**, Ω*_i_*) to the total electron density deriving from that atom:
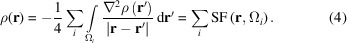
An atomic basin can yield a positive or a negative contribution to ρ(**r**), therefore behaving as either a ‘source’ or a ‘sink’ of electron density, respectively.

Starting from QTAIM, the IQA (Blanco *et al.*, 2005 ▸; Francisco *et al.*, 2006 ▸; Guevara-Vela *et al.*, 2020 ▸) provides a general energy partition scheme to decompose the total energy of a system into atomic and interatomic contributions:
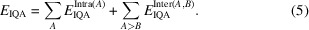
Atomic contributions account for the kinetic energy and the electron–nucleus and electron–electron interactions for particles belonging to the same atom:
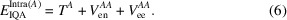
Interatomic energy, on the other hand, includes all the interactions between each atom pair (*i.e.* nucleus–nucleus, nucleus–electron and electron–electron interactions):
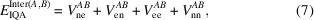
where the electron–electron term comprises a Coulomb part 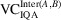 and an exchange-correlation part 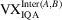. The interatomic term 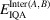 therefore represents only a contribution to the total binding energy, 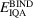, between two interacting molecular fragments F_1_ and F_2_. This latter term, in fact, accounts not only for all the interactions between atom pairs belonging to each molecular fragment, 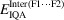, but also for both atomic, 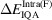, and interatomic, 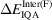, energy variations upon complexation inside each fragment (Syzgantseva *et al.*, 2013 ▸):
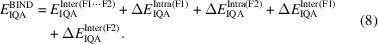
Based on these formulae, IQA provides both the ‘two-centre’ XB energy [*i.e.* the interaction energy between the XB donor (*X*) and acceptor (*A*)], 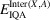, and the ‘total’ XB energy, 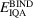. The latter is expected to recover the ‘conventional’ binding energy, 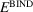, computed as the difference between the energies of the complex and the isolated fragments, in particular when dispersion contributions are small (Syzgantseva *et al.*, 2013 ▸).

## 3. Computational details

*Ab initio* calculations were performed using the *Gaussian16* software (Frisch *et al.*, 2016 ▸). The systems were investigated at both MP2 (Head-Gordon & Head-Gordon, 1994 ▸; Saebø & Almlöf, 1989 ▸; Head-Gordon *et al.*, 1988 ▸; Frisch *et al.*, 1990*a* ▸,*b* ▸) and DFT levels of theory, employing the ubiquitously used B3LYP functional (Vosko *et al.*, 1980 ▸; Lee *et al.*, 1988 ▸; Becke, 1993 ▸) and four other functionals: M06-2X (Zhao & Truhlar, 2008 ▸), M11 (Peverati & Truhlar, 2011 ▸), ωB97X (Chai & Head-Gordon, 2008*b* ▸) and ωB97XD (Chai & Head-Gordon, 2008*a* ▸), which gave a better performance according to our previous QTAIM investigation on XBs (Forni *et al.*, 2016 ▸). All calculations were performed with the 6-311++G(d,p) basis set. For the iodine atom, this basis set, not internally stored in *Gaussian16*, was built up by downloading the 6-311G(d,p) one from the basis set exchange site (Feller, 1996 ▸; Schuchardt *et al.*, 2007 ▸) and retrieving the diffuse functions from the literature (Glukhovtsev *et al.*, 1995 ▸).

Geometry optimizations were carried out at the DFT level using an ultrafine grid with 99 radial shells and 590 angular points per shell. All minima have been confirmed by vibrational frequency analysis. Due to the high computational cost of the MP2 method, we have used the complexes at fixed geometry obtained at the B3LYP/6-1++G(d,p) level. On each minimum-energy structure, for every above-mentioned method, a single-point calculation was performed to introduce the counterpoise correction and obtain the binding energy, *E*^BIND^, computed as the difference in energy between the complex and the isolated non-relaxed fragments.

The wavefunctions obtained by optimization at the DFT level, and by single-point calculations without the counterpoise correction at the MP2 level, have been used for QTAIM and IQA analyses by means of the *AIMAll* software (Todd, 2019 ▸). In particular, we computed the electron density values, ρ(**r**_BCP_), and the atomic contributions to the source function, SF(Ω), at the bond critical point (BCP) of the *X*⋯N XB, the latter quantity also expressed as percentage contributions, SF%(Ω), to the total density in the reference point, ρ(**r**_BCP_). The delocalization indices δ(Ω_*X*_, Ω_N_) have also been evaluated.

The two-centre XB energy, 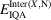, and its exchange-correlation contribution, 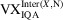, have been computed at the B3LYP, M06-2X and MP2 levels of theory, all of them supported in *AIMAll*. Note that quantities derived from the second-order density matrix, such as delocalization indices and the two-atomic exchange energies in IQA, are not rigorously defined within DFT, preventing, in principle, their exact evaluation. However, suitable approximations have been implemented in *AIMAll* allowing, in particular, the total energy of the system to be recovered from the IQA components (Maxwell *et al.*, 2016 ▸). Concerning the MP2 decomposition, *AIMAll* implementation of IQA analysis uses natural orbitals of the one-electron density matrix to compute the two-electron density matrix through the Müller approximation (Müller, 1984 ▸). The extent of this approximation has been previously tested (Tognetti *et al.*, 2018 ▸) by comparison with that implemented in *Morphy*, which uses Hartree–Fock orbitals and provides the exact MP2 IQA partitioning (McDonagh *et al.*, 2016 ▸; Popelier, 1996 ▸). Evaluation of the three (kinetic, electrostatic and exchange-correlation) physical contributions to atomic energies through the two approaches revealed that, for light-atom (*i.e.* C, N, O and F) containing molecules, the Müller approximation provides systematically more positive kinetic energies and more negative electrostatic and exchange-correlation energies (Tognetti *et al.*, 2018 ▸). It is therefore argued that relating DFT and MP2 properties obtained through different IQA implementations is completely meaningless, while reliable conclusions can be drawn when comparing quantities obtained for a series of compounds within a given approach.

The 2D contour diagrams of the integrand of the SF have been generated with *Multiwfn* (version 3.8; Lu & Chen, 2012 ▸) using **r**_BCP_ as the reference point. Finally, molecular representations have been obtained with the *Gaussview* software (Dennington *et al.*, 2019 ▸).

## 4. Results and discussion

Based on the work of Bartashevich *et al.* (2014 ▸), we have considered, as XB acceptors, the same set of substituted pyridines (*R*-py, *i.e.* 15 pyridine-based compounds bearing different electron-withdrawing and electron-donor substituents) located in different positions on the ring. The presence of substituents in position 2 allows the evaluation of combined effects arising from electronic factors and steric hindrance with the approaching halogen atom. As XB donors, in addition to I_2_ (Bartashevich *et al.*, 2014 ▸), other halogenated molecules (*i.e.* Br_2_, ICN and BrCN) have been considered to evaluate the effects of both the nature of the halogen and the attached substituent on the properties of the σ-hole and the XB features. In line with the investigation of Bartashevich *et al.* (2014 ▸), the B3LYP functional has been adopted throughout. This functional was demonstrated to provide the most accurate IQA energies, using coupled cluster singles and doubles (CCSD) data as a reference, in a previous investigation on intramolecular interactions in glycol conformers (Cukrowski, 2019 ▸). Selected calculations have also been performed at the MP2 and DFT levels, using four other functionals besides B3LYP for the latter, to highlight the differences between energies, QTAIM properties and IQA results obtained at different levels of theory.

**Figure 1:**
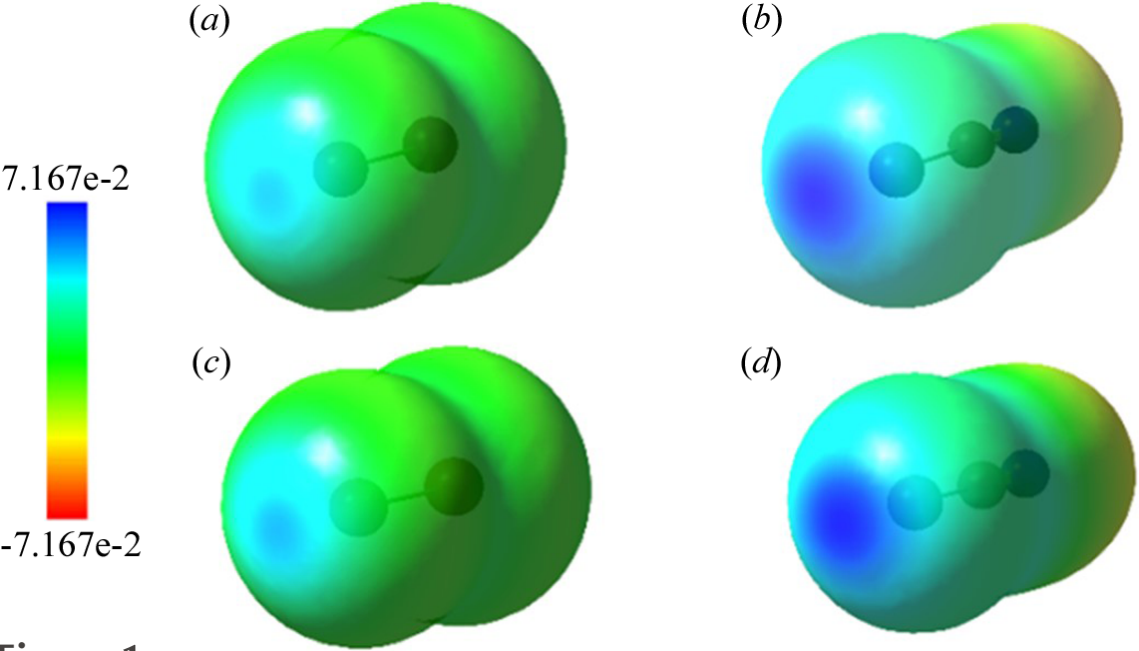
ESPs on the isosurface of electron density (0.001 electrons bohr^−3^) calculated at the B3LYP/6-311++G(d,p) level for (*a*) I_2_, (*b*) ICN, (*c*) Br_2_ and (*d*) BrCN.

In Tables S1–S6 of the supporting information, the computed interaction energies (*E*^BIND^) obtained at the B3LYP, MP2, M06-2X, M11, ωB97X and ωB97XD levels for the four *DX*⋯(*R*-)py series of complexes (*DX* = I_2_, Br_2_, ICN and BrCN) are reported. The substituted pyridines are sorted according to the scale of experimental values of p*K*B_I_2__ (Table S7), where p*K*B_I_2__ = log_10_[*K*_c_], *K*_c_ being the equilibrium constant for the reaction py + I_2_ → py⋯I—I in hexane at 298 K (Laurence *et al.*, 2011 ▸). Binding energies, approximately increasing in the same order as the p*K*B_I_2__ values, denote the formation of XBs of medium strength (Cavallo *et al.*, 2016 ▸). Interestingly, considering the MP2 reference values, the *E*^BIND^ of complexes with I_2_ (−6.3 to −12.1 kcal mol^−1^) fall into approximately the same range as those of Br_2_ (−6.0 to −12.3 kcal mol^−1^), differing from the generally reported increase of XB strength with halogen weight. However, note that most computational studies on XBs involving *X*_2_dihalogens focus on the lighter atoms: F_2_, Cl_2_ and Br_2_ (Alkorta *et al.*, 1998 ▸; Wang *et al.*, 2014 ▸; Karpfen, 2003 ▸) with only few exceptions including I_2_ (Bartashevich *et al.*, 2015 ▸; McAllister *et al.*, 2023 ▸). These results suggest that further assessment of the XB strength involving the full series of dihalogens using, in particular, well calibrated basis sets, is highly desirable. Replacement of the non-interacting halogen with the electron-withdrawing CN group implies a decrease of *E*^BIND^ in almost all cases, which is much more pronounced for bromine complexes, restoring the expected I > Br energy trend. MP2 results therefore show that *E*^BIND^ decreases in the order I_2_ ≃ Br_2_ > ICN > BrCN. Note, however, that *X*CN molecules are characterized by larger and higher σ-holes than those of the corresponding *X*_2_ dihalogens (Fig. 1 ▸), as expected based on the different electronegativity of the involved species. More precisely, the maximum ESP values at the σ-holes are +0.031, +0.034, +0.056 and +0.055 a.u. for I_2_, Br_2_, ICN and BrCN, respectively.

These results provide evidence that halogen bonding is not (always) dominated by electrostatics, because the concept of the σ-hole cannot be considered as the single parameter to be associated with the strength of the interaction (Tognetti & Joubert, 2016 ▸). As recognized by Tognetti and coworkers (Syzgantseva *et al.*, 2013 ▸): ‘at long-range, electrostatics clearly dominate (as expected from the σ-hole model) and are responsible for the initiation of the bond formation process’. At short range, covalence contributions can become important, as demonstrated by the VB/MO (Wang *et al.*, 2014 ▸), SCVB (Franchini *et al.*, 2019 ▸; Forni *et al.*, 2024 ▸) and MP4/IQA calculations (Alkorta *et al.*, 2020 ▸).

Looking at the DFT results obtained with the different examined functionals, the better agreement with MP2 is provided by M06-2X calculations, confirming this functional as one of the best for modelling XB complexes at the DFT level (Kozuch & Martin, 2013 ▸; Forni *et al.*, 2016 ▸, 2014 ▸, 2012 ▸), though all the other functionals display an acceptable performance.

IQA studies have been performed on MP2 and DFT wavefunctions (within the approximations adopted in *AIMAll*, see *Computational details*[Sec sec3]) using, for the latter, the B3LYP and M06-2X functionals. In particular, owing to its relatively affordable computational costs, B3LYP has been used to determine the total binding energy (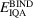) according to equation (8), and ascertain the error, due to the adopted approximations, in recovering the exact B3LYP 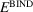 value (see *Computational details*[Sec sec3]). The relative errors, reported in Table S9, are always below 10% for the complexes with bromine and below 15% for those with iodine, though no systematic trends can be individuated. Note that the rather good agreement between the two energy values (see Fig. S1 of the supporting information) is due to the relatively high interaction energies of the investigated systems, where dispersion contributions are low (Syzgantseva *et al.*, 2013 ▸).

The two-centre XB energies 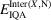 [see equation (7)] obtained at the B3LYP, MP2 and M06-2X levels are reported in Table S10. Analysis of a possible correlation between 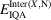 and *E*^BIND^ for each *DX*⋯(*R*-)py set indicates that, in general, these descriptors are not correlated for the present systems, except for only a few fortuitous cases (Fig. S2). This result indicates that the formation of medium-strength XBs implies a strong rearrangement within the two interacting fragments, with associated variations of atomic energies which depend on both the *DX* and the *R*-py species (Tognetti & Joubert, 2016 ▸). Note that, in the case of strong XB interactions (*e.g.* involving anions), the intra-fragment energy variations become less relevant with respect to the interaction energy, resulting in good agreement between *E*^BIND^ and 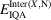 (Syzgantseva *et al.*, 2013 ▸).

Inspection of the percentage contribution of the exchange-correlation energy, 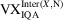, to 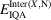 (Table S10) indicates that this term always has a remarkable weight, in some cases comparable to or even greater than the electrostatic 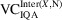 one. We note that:

(i) For each *DX* donor, such contribution shows only a slight increase with the strength of the interaction, from about 3 to 6% according to the method and the *DX* donor, in spite of a much greater increase of 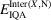, allowing us to consider an average contribution for each set of complexes, as reported in Table 1 ▸. Here, it can be observed that the B3LYP functional systematically provides the greater exchange-correlation contributions, followed by MP2 and then M06-2X.

(ii) Complexes with iodo-derivatives have much greater 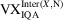 contributions than the corresponding bromo-derivative complexes.

(iii) For systems containing *X*CN, 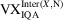 is significantly lower with respect to systems containing *X*_2_, in agreement with the electron-withdrawing power of the CN group, which imparts greater electrostatic character in the interaction. Accordingly, the average 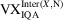 values are found to increase in the order Br_2_ < BrCN < I_2_ < ICN or, equivalently, the electrostatic contribution, 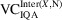, to the 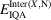 interaction energy decreases in the same order from ICN to Br_2_, as qualitatively inferred from the electronegativity of the atomic species involved.

**Figure 2:**
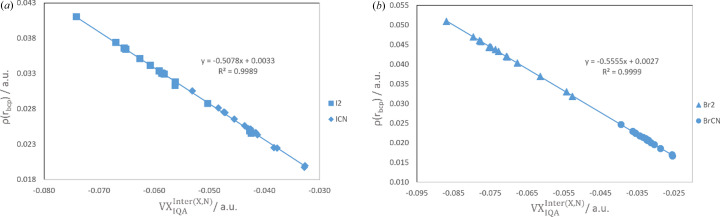
Relationships between electron density at the *X*⋯N BCP, ρ(**r**_BCP_) and exchange-correlation contribution to the XB energy obtained at the B3LYP level of theory for complexes formed with (*a*) iodine and (*b*) bromine derivatives.

The existence of possible relationships between the two-atomic energy contributions and local properties of the electron density has been then tested. The exchange-correlation contribution was found to correlate very well, at each level of theory, with both the electron density at the BCP of the *X*⋯N bond, ρ_*X*⋯N_(**r**_BCP_), and the delocalization index δ(Ω_*X*_, Ω_N_). The relationships are found to depend only on the nature of the halogen atom. The correlations of ρ_*X*⋯N_(**r**_BCP_) with 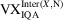 obtained at the B3LYP level are reported in Fig. 2 ▸ [see Figs. S3 and S4 for all results obtained at the B3LYP, MP2 and M06-2X levels for the correlations of ρ_*X*⋯N_(**r**_BCP_) and δ(Ω_X_, Ω_N_), respectively, with 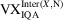].

**Figure 3:**
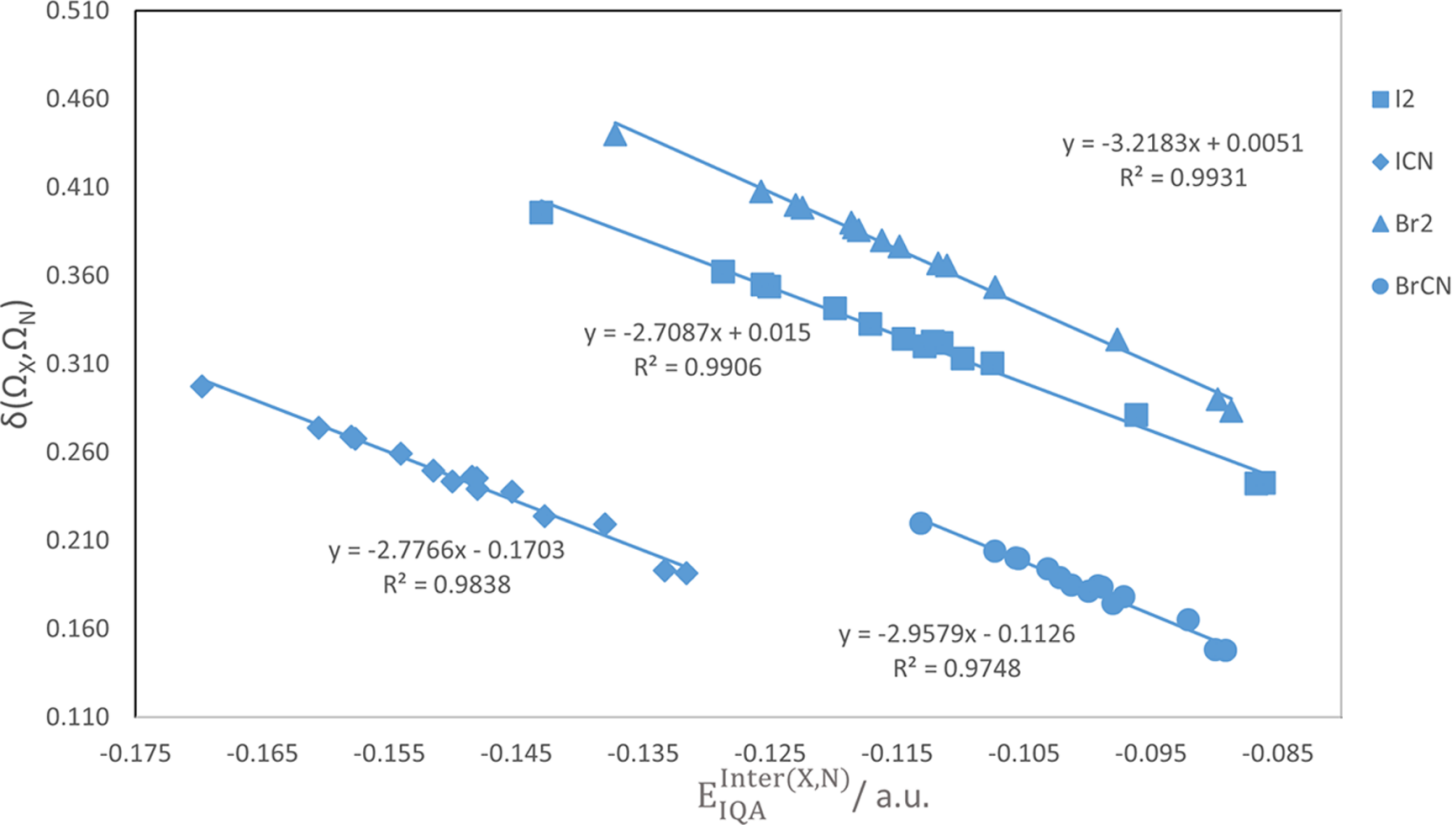
Relationships between the delocalization index and the IQA XB energy obtained at the B3LYP level.

Moreover, a good correlation of 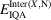 with both ρ_X⋯N_(**r**_BCP_) and δ(Ω_*X*_, Ω_N_) was also found at each level of theory but, in this case, four different relationships have been identified, one for each set of dimers. In Fig. 3 ▸ the correlations of 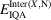 with δ(Ω_*X*_, Ω_N_) obtained at the B3LYP level are reported (see Figs. S5 and S6 for the whole set of results). Note that, for both I⋯N and Br⋯N interactions, complexes formed with dihalogens display better correlation than those with *X*CN.

On the other hand, no correlation has been found between 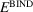 and δ(Ω_*X*_, Ω_N_) (Fig. S7). More precisely, for each dataset, only 9 out of 15 complexes show a linear trend. The dimers deviating from this trend correspond to the *ortho*-substituted pyridines, whose deviations increase with the size of the substituent. Moreover, it is greater for complexes with *X*CN, suggesting the presence of interactions between the substituent on the pyridine ring and both the halogen and its attached CN group. This result underlines the importance of secondary interactions on the stability of the complexes, which cannot be predicted by simply analysing the properties exclusively connected to the interacting *X* and N atoms (Syzgantseva *et al.*, 2013 ▸).

**Figure 4:**
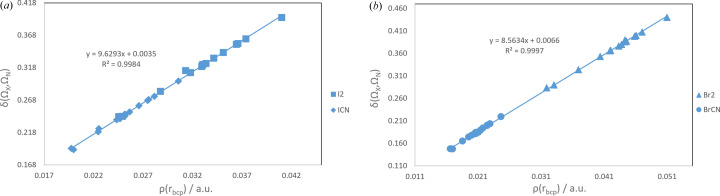
Relationships between the δ(Ω_*X*_, Ω_N_) delocalization index and the electron density at the *X*⋯N BCP obtained at the B3LYP level for complexes formed with moieties containing (*a*) iodine and (*b*) bromine atoms.

Finally, halogen-dependent relationships have also been found between ρ_*X*⋯N_(**r**_BCP_) and δ(Ω_*X*_, Ω_N_). Fig. 4 ▸ illustrates how the delocalization index between the two basins is proportional to the electron density at the *X*⋯N BCP. All results are collected in Fig. S8.

Further information on the nature of the *X*⋯N XB is provided by the SF in the corresponding **r**_BCP_. Looking at Tables S1–S6, reporting the absolute (SF) and percentage (SF%) values of the SF for halogen and nitro­gen atoms, the main contribution to ρ_*X*⋯N_(**r**_BCP_) as a source of electron density (*i.e.* with positive SF) comes from the halogen, with SF% values up to 50%, and slightly increasing with the strength of the interaction and decreasing in the order I_2_ ≃ Br_2_ > ICN > BrCN. The nitro­gen atom displays a greater variability in its SF% contribution, acting as either a sink (in *X*CN complexes) or a source (in most *X*_2_ ones) to ρ_*X*⋯N_(**r**_BCP_). Again, the stronger the interaction, the greater the nitro­gen SF% value, the maximum value being 16% (B3LYP and MP2 results). Remarkably, the sum of SF% on the halogen and nitro­gen atoms accounts for far less than 100% of the electron density at the *X*⋯N BCP, their cumulative contributions amounting at most to 65% (in Br_2_ complexes, B3LYP and MP2 results). The remaining density mainly comes from the atoms directly bonded to the interacting *X* and N atoms and gradually decreases moving away from them, therefore evidencing how the *X*⋯N BCP topological properties are strongly influenced by the distal atoms.

**Figure 5:**
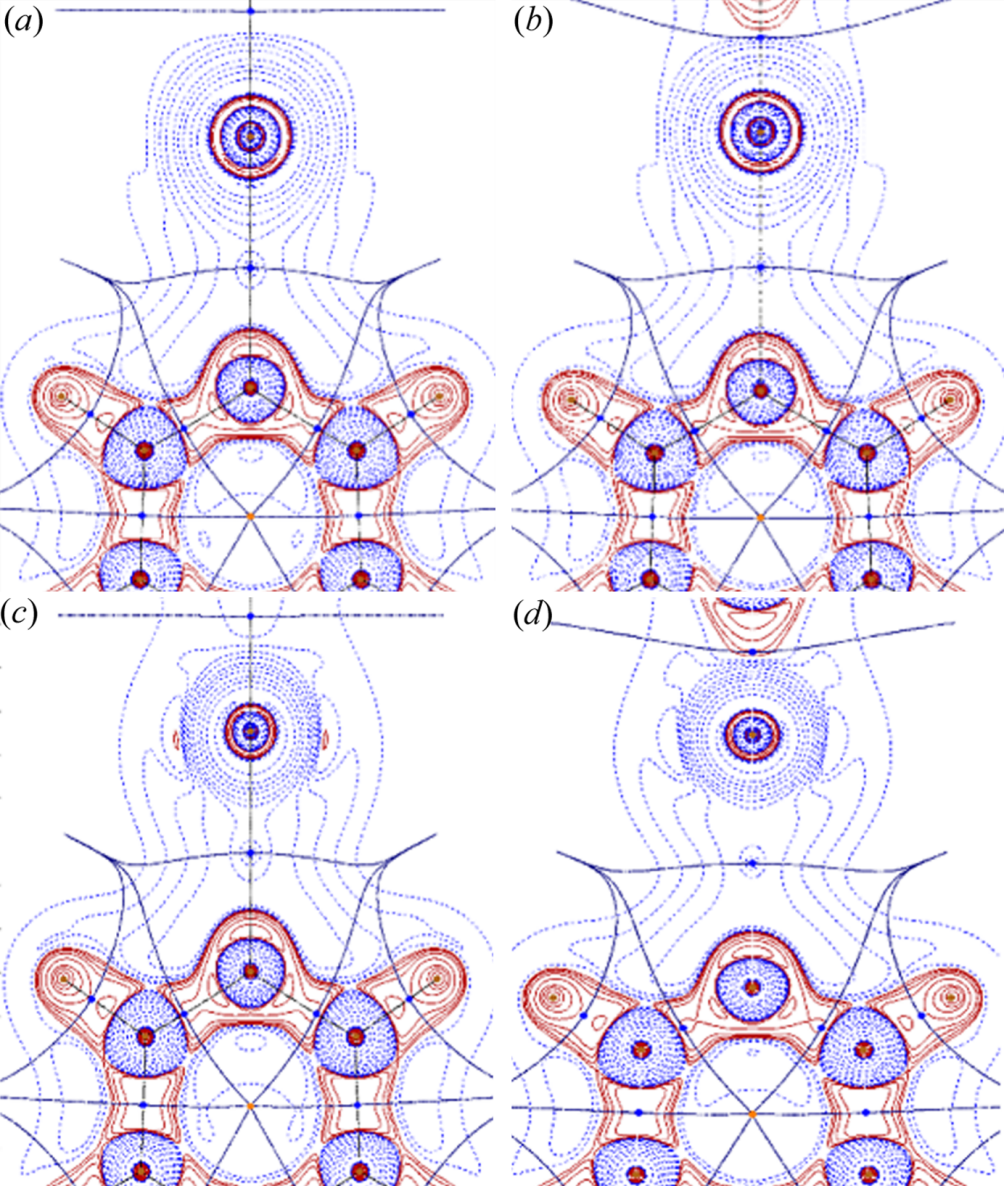
2D contour diagrams of *L*(**r**) for the complexes of pyridine with (*a*) I_2_, (*b*) ICN, (*c*) Br_2_ and (*d*) BrCN using the B3LYP wavefunctions. Solid-red and dashed-blue lines correspond to positive and negative contours, respectively.

The different atomic contributions to electron density at the ρ_*X*⋯N_(**r**_BCP_) can of course be visually inspected by looking at the integrand of the SF, 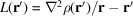. The 2D contour diagrams of *L*(**r**) for the complexes of unsubstituted pyridine with I_2_, ICN, Br_2_ and BrCN using the B3LYP wavefunctions are reported in Fig. 5 ▸, focusing on the XB region. By comparing the *L*(**r**) contour diagrams of the complexes with I_2_ and Br_2_ [Figs. 5 ▸(*a*) and 5 ▸(*c*)], it is clear that the bromine atom contributes more significantly than the iodine atom to ρ_*X*⋯N_(**r**_BCP_), explaining the higher interaction energy associated with the (Br-)Br⋯N bond in this complex. When the halogen bonded to bromine is substituted with a CN group, the bromine contribution at the BCP decreases [Fig. 5 ▸(*d*)]. A comparable effect is observed for its iodo-derivative analogue, though the extent of this decrease is less pronounced [Fig. 5 ▸(*b*)], reflecting in the higher interaction energy for the complex with ICN with respect to that with BrCN.

**Figure 6:**
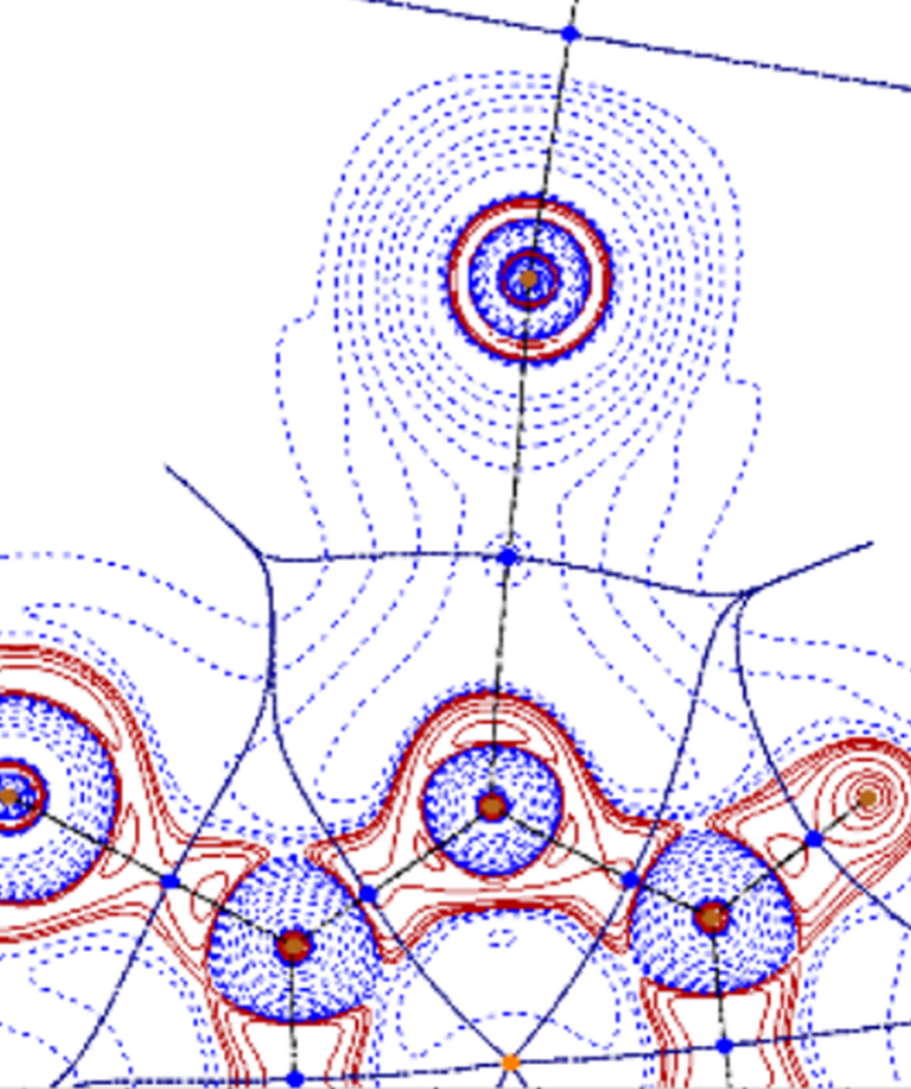
2D contour diagrams of *L*(**r**) for the complex 2-chloro­pyridine·I_2_ using the B3LYP wavefunction. Solid-red and dashed-blue lines correspond to positive and negative contours, respectively.

When pyridine bears substituents in position 2, a drop in the SF% contribution for both the halogen and the nitro­gen atoms is observed. This result can be explained by looking at the corresponding *L*(**r**) contour diagrams. Comparing the plot for the complex 2-chloro­pyridine·I_2_ (Fig. 6 ▸) with that of pyridine·I_2_ [Fig. 5 ▸(*a*)], we can see that the presence of the chlorine atom, besides determining a deviation of the C– I⋯N bond angle from linearity, influences the shape and the extension of both the nitro­gen and iodine basins, reducing their contribution to *L*(**r**). Concomitantly, a high SF% value (8%) is obtained for the chlorine atom, much greater than that of the hydrogen atom (4%) placed in the same position in pyridine·I_2_. Analogous results have been obtained replacing chlorine with different groups such as -F, -CH_3_, -CH_2_CH_3_ and -CH(CH_3_)_2_.

The lower extension of nitro­gen and halogen basins in the 2-substituted complexes is associated with a lower delocalization index between them. It was previously demonstrated that, for the (I-)I⋯N XB (Bartashevich *et al.*, 2014 ▸), the corresponding delocalization index is quantitatively correlated to the sum of the atomic contributions of the halogen and nitro­gen atoms to ρ_*X*⋯N_(**r**_BCP_). We can generalize this finding by expressing 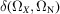 as
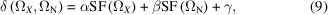
with different α, β and γ values according to both the different set of complexes and the level of theory, as summarized in Table S11. A very good agreement is observed between the δ(Ω_*X*_, Ω_N_) values (Tables S1–S6) and those calculated with equation (9), reported in Table S12.

It is also interesting to observe how substituents in positions 3 and 4 influence the electron density at the BCP. Halogen and nitro­gen contributions to the SF are greater, compared with those obtained with non-substituted pyridine, for systems containing electron-donor groups, while a drop is observed for those containing electron-withdrawing groups. Moreover, the electron-donation effect increases with the size of the substituent, but it appears that increasing the number of substituents is preferable to increasing their size. All these variations in the atomic contributions to ρ_*X*⋯N_(**r**_BCP_) clearly reflect in different interaction energies, allowing us to quantitatively relate the strength of the interaction to the specific SF% values of both the interacting atomic pair and the distal atoms.

## 5. Conclusions

The *X*⋯N (*X* = I, Br) halogen-bonding interactions in complexes formed by substituted pyridines and *X*CN/*X*_2_ molecules have been investigated in the QTAIM/IQA framework. IQA analysis underlines the importance of the exchange-correlation contribution to the total interaction energy, in some cases dominating the electrostatic term. Quantitative relationships between IQA energies and QTAIM descriptors, such as the electron density at the *X*⋯N BCP and delocalization index, have been found. Such topological properties generally show a good correlation with the two-centre IQA interaction energy between the two main atoms involved in the interaction. The obtained relationships only depend on the nature of the halogen atom if the sole exchange-correlation contribution is considered. On the other hand, no correlations with local topological properties at the *X*⋯N BCP are in general found if the total binding energies are considered, underlying the important role of the local environment on the stability of the complexes. This point is quantified by analysis of the atomic contributions to the electron density at the *X*⋯N BCP, as provided by the SF. This analysis allows us to show the essentially non-local character of electron density and quantitatively relates the strength of the interaction with specific atomic contributions coming from, not only the directly interacting pair, but also the distal atoms including the different substituents on the pyridine ring.

## Supplementary Material

Supporting figures and tables. DOI: 10.1107/S2052252525000363/pen5006sup1.pdf

## Figures and Tables

**Table 1:** Average percentage contribution of the exchange-correlation term, 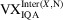, to the two-centre XB energy, 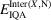, computed at the B3LYP, MP2 and M06-2X levels of theory

	B3LYP	MP2	M06-2X
I_2_	51.60	49.01	44.09
Br_2_	28.37	27.59	25.13
ICN	62.86	57.98	55.97
BrCN	32.14	30.05	29.05

## Data Availability

The data supporting the results reported in this article can be accessed within the article and as published supporting material.
