# Supplementary material for: The nature of halogen bonding: insights from interacting quantum atoms and source function studies
Source: IUCrJ. 2025 Jan 27;12(Pt 2):188–97. doi: 10.1107/S2052252525000363 (PMC11878453; doi:10.1107/S2052252525000363)
Supplement: Supplementary file 1 [file m-12-00188-sup1.pdf]

IUCrJ

Volume 12 (2025)

Supporting information for article:

The nature of halogen bonding: Insights from Interacting Quantum Atoms and source function studies

Arianna Pisati, Alessandra Forni, Stefano Pieraccini and Maurizio Sironi

The nature of halogen bonding: Insights from Interacting Quantum Atoms and source function studies

Arianna Pisati,[1,2] Alessandra Forni,[3,*] Stefano Pieraccini,[2,3*] and Maurizio Sironi[2,3,*]

1 Department of Pharmaceutical Sciences, Università degli Studi di Milano, via Mangiagalli 25, 20133 Milano, Italy

2 Department of Chemistry, Università degli Studi di Milano, via Golgi 19, 20133 Milano, Italy

3 CNR-SCITEC, Institute of Chemical Sciences and Technologies "Giulio Natta" and INSTM RU, via Golgi 19, 20133 Milano, Italy

Supporting information

Table S1 B3LYP values of E_{BIND} (kcal/mol), $\rho_{X\cdots N}(\mathbf{r}_{bcp})$ (a.u.), absolute (SF) and percentage (SF%) halogen and nitrogen contributions (a.u.) to $\rho_{X\cdots N}(\mathbf{r}_{bcp})$ and delocalization indices between halogen and nitrogen, $\delta(\Omega_X,\Omega_N)$, in the set of complexes of substituted pyridines with I_2, ICN, Br_2 and BrCN molecules.

Complex	E_{BIND}	$\rho_{X\cdots N}(\mathbf{r}_{bcp})$	SF(Ω_X)	SF(Ω_N)	SF%(Ω_X)	SF%(Ω_N)	$\delta(\Omega_X, \Omega_N)$
2-Fluoropyridine·I_2	-4.72	0.024532	0.011454	-0.002066	46.663403	-8.416943	0.242515
2-Chloropyridine·I_2	-4.69	0.024905	0.011488	-0.002010	46.190586	-8.082409	0.242615
3,5-Dichloropyridine·I_2	-5.28	0.028754	0.013806	-0.000450	48.014384	-1.565779	0.281321
3-Chloropyridine·I_2	-6.49	0.031806	0.015455	0.000742	48.575855	2.333482	0.310402
3-Acetylpyridine·I_2	-6.96	0.033001	0.016128	0.001240	48.837922	3.756167	0.321859
2-Isopropylpyridine·I_2	-7.55	0.032923	0.015743	0.000981	47.759606	2.977531	0.320002
4-Acetylpyridine·I_2	-6.94	0.033062	0.016163	0.001272	48.864306	3.844137	0.322760
2-Ethylpyridine·I_2	-7.63	0.033373	0.015892	0.001213	47.716661	3.641291	0.324338
Pyridine·I_2	-8.01	0.035116	0.017259	0.002017	49.171782	5.746153	0.341516
2,4,6-Trimethylpyridine·I_2	-7.33	0.031304	0.014681	0.000529	46.851127	1.688216	0.313272
2-Methylpyridine·I_2	-7.87	0.034137	0.016560	0.001506	48.470240	4.407560	0.332672
4-Methylpyridine·I_2	-8.60	0.036428	0.017991	0.002502	49.401389	6.870632	0.353812
4-Ethylpyridine·I_2	-8.65	0.036585	0.018118	0.002579	49.461261	7.039503	0.355164
3,4-Dimethylpyridine·I_2	-9.05	0.037394	0.018557	0.002806	49.570134	7.494422	0.362340
4-Dimethylamminopyridine·I_2	-10.81	0.041021	0.020559	0.004253	50.123524	10.369503	0.395801
2-Fluoropyridine·ICN	-5.58	0.019746	0.007917	-0.003565	40.131760	-18.070474	0.193325
2-Chloropyridine·ICN	-5.46	0.019964	0.007834	-0.003518	39.345858	-17.666804	0.191746
3,5-Dichloropyridine·ICN	-5.34	0.022462	0.009389	-0.002437	41.813908	-10.854555	0.219221
3-Chloropyridine·ICN	-6.41	0.024334	0.010368	-0.001682	42.565212	-6.904812	0.237663
3-Acetylpyridine·ICN	-6.65	0.025175	0.010734	-0.001319	42.734428	-5.251459	0.246217
2-Isopropylpyridine·ICN	-7.11	0.024661	0.010297	-0.001745	41.701911	-7.066490	0.239208
4-Acetylpyridine·ICN	-6.74	0.025094	0.010792	-0.001355	42.951736	-5.392712	0.245569

IMPORTANT: this document contains embedded data - to preserve data integrity, please ensure where possible that the IUCr Word tools (available from http://journals.iucr.org/services/docxtemplate/) are installed when editing this document.

2-Ethylpyridine·ICN	-7.18	0.025165	0.010578	-0.001526	41.786208	-6.027198	0.243553
Pyridine·ICN	-7.69	0.026575	0.011576	-0.000871	43.578220	-3.279381	0.259403
2,4,6-Trimethylpyridine·ICN	-6.79	0.022523	0.009021	-0.002393	39.970457	-10.600944	0.223939
2-Methylpyridine·ICN	-7.42	0.025637	0.010913	-0.001379	42.603815	-5.382118	0.249541
4-Methylpyridine·ICN	-8.20	0.027457	0.012019	-0.000520	43.768781	-1.894270	0.267895
4-Ethylpyridine·ICN	-8.24	0.027556	0.012113	-0.000478	43.881726	-1.730616	0.268769
3,4-Dimethylpyridine·ICN	-8.56	0.028131	0.012321	-0.000322	43.884101	-1.146924	0.273949
4-Dimethylamminopyridine·ICN	-10.02	0.030560	0.013711	0.000619	44.868017	2.026024	0.297431
2-Fluoropyridine·Br_2	-6.17	0.031879	0.014667	0.000452	46.027662	1.419432	0.283581
2-Chloropyridine·Br_2	-6.34	0.033056	0.015054	0.000858	45.666347	2.602082	0.289863
3,5-Dichloropyridine·Br_2	-7.15	0.036911	0.017438	0.002472	47.247760	6.697419	0.323993
3-Chloropyridine·Br_2	-8.62	0.040379	0.019281	0.003908	47.747299	9.677392	0.353322
3-Acetylpyridine·Br_2	-9.25	0.041905	0.020154	0.004534	48.079034	10.815715	0.365907
2-Isopropylpyridine·Br_2	-10.47	0.043315	0.020545	0.004857	47.416977	11.208766	0.376428
4-Acetylpyridine·Br_2	-9.20	0.042009	0.020200	0.004613	48.045637	10.970740	0.367061
2-Ethylpyridine·Br_2	-10.55	0.043767	0.020709	0.005133	47.366082	11.740622	0.380178
Pyridine·Br_2	-10.46	0.044283	0.021389	0.005479	48.320275	12.377753	0.385771
2,4,6-Trimethylpyridine·Br_2	-11.01	0.044307	0.020893	0.005337	47.111258	12.035581	0.389834
2-Methylpyridine·Br_2	-10.76	0.044521	0.021353	0.005329	48.054167	11.991545	0.387225
4-Methylpyridine·Br_2	-11.14	0.045813	0.022264	0.006027	48.637361	13.167227	0.398335
4-Ethylpyridine·Br_2	-11.21	0.046046	0.022409	0.006189	48.598175	13.423305	0.400118
3,4-Dimethylpyridine·Br_2	-11.69	0.047007	0.022870	0.006493	48.649685	13.811040	0.407714
4-Dimethylamminopyridine·Br_2	-13.68	0.050948	0.025014	0.008164	49.105146	16.025682	0.440154
2-Fluoropyridine·BrCN	-4.61	0.016616	0.005355	-0.004568	32.280682	-27.539231	0.148071
2-Chloropyridine·BrCN	-4.51	0.016968	0.005364	-0.004478	31.696194	-26.461883	0.147638
3,5-Dichloropyridine·BrCN	-4.27	0.018533	0.006355	-0.003699	34.306401	-19.970327	0.164986
3-Chloropyridine·BrCN	-5.09	0.020014	0.007100	-0.003108	35.450071	-15.517466	0.178308
3-Acetylpyridine·BrCN	-5.22	0.020670	0.007337	-0.002807	35.594437	-13.618111	0.184419
2-Isopropylpyridine·BrCN	-5.65	0.020653	0.007239	-0.003087	34.913087	-14.888160	0.181408
4-Acetylpyridine·BrCN	-5.31	0.020567	0.007387	-0.002848	35.867167	-13.826611	0.183587
2-Ethylpyridine·BrCN	-5.69	0.021055	0.007459	-0.002943	35.339345	-13.945062	0.184609
Pyridine·BrCN	-6.05	0.021761	0.007987	-0.002476	36.723833	-11.385639	0.193873
2,4,6-Trimethylpyridine·BrCN	-5.59	0.019525	0.006515	-0.003462	33.380700	-17.740133	0.174176
2-Methylpyridine·BrCN	-5.88	0.021381	0.007661	-0.002791	35.897180	-13.077878	0.188892
4-Methylpyridine·BrCN	-6.41	0.022403	0.008307	-0.002258	37.142550	-10.094458	0.199633
4-Ethylpyridine·BrCN	-6.44	0.022473	0.008359	-0.002187	37.117811	-9.712056	0.200203
3,4-Dimethylpyridine·BrCN	-6.66	0.022895	0.008480	-0.002087	37.131170	-9.137211	0.203724
4-Dimethylamminopyridine·BrCN	-7.68	0.024638	0.009432	-0.001446	38.349004	-5.877986	0.219543

Table S2 M06-2X values of E_{BIND} (kcal/mol), $\rho_{X\cdots N}(\mathbf{r}_{bcp})$ (a.u.), absolute (SF) and percentage (SF%) halogen and nitrogen contributions (a.u.) to $\rho_{X\cdots N}(\mathbf{r}_{bcp})$ and delocalization indices between halogen and nitrogen, $\delta(\Omega_X,\Omega_N)$, in the set of complexes of substituted pyridines with I_2, ICN, Br_2 and BrCN molecules.

Complex	E_{BIND}	$\rho_{X\cdots N}(\mathbf{r}_{bcp})$	SF(Ω_X)	SF(Ω_N)	SF%(Ω_X)	SF%(Ω_N)	$\delta(\Omega_X, \Omega_N)$
2-Fluoropyridine·I_2	*-6.11*	*0.022777*	*0.010370*	*-0.002850*	*45.524531*	*-12.510155*	*0.210586*

2-Chloropyridine·I_2	-6.59	0.023776	0.010782	-0.002611	45.287391	-10.967923	0.215077
3,5-Dichloropyridine·I_2	-6.18	0.026050	0.012202	-0.001570	46.839376	-6.026921	0.239975
3-Chloropyridine·I_2	-7.20	0.029626	0.014158	-0.000208	47.734071	-0.700575	0.271914
3-Acetylpyridine·I_2	-7.61	0.031344	0.015094	0.000483	48.093752	1.537527	0.287221
2-Isopropylpyridine·I_2	-9.44	0.032522	0.015308	0.000581	46.953207	1.782543	0.291707
4-Acetylpyridine·I_2	-7.59	0.030931	0.014867	0.000337	48.030301	1.087879	0.283993
2-Ethylpyridine·I_2	-9.49	0.033226	0.015641	0.000902	47.148207	2.720413	0.299013
Pyridine·I_2	-8.55	0.034031	0.016539	0.001446	48.632087	4.25261	0.310699
2,4,6-Trimethylpyridine·I_2	-9.81	0.031264	0.014426	0.000276	46.166879	0.883107	0.287173
2-Methylpyridine·I_2	-9.30	0.034201	0.016428	0.001298	48.050702	3.795072	0.309662
4-Methylpyridine·I_2	-9.06	0.035909	0.017618	0.002102	49.092942	5.856833	0.327062
4-Ethylpyridine·I_2	-9.10	0.036065	0.017703	0.002187	49.069814	6.061906	0.328499
3,4-Dimethylpyridine·I_2	-9.46	0.037273	0.018363	0.002595	49.230414	6.956003	0.338507
4-Dimethylamminopyridine·I_2	-11.12	0.042452	0.021224	0.004637	49.999469	10.923659	0.383408
2-Fluoropyridine·ICN	-7.58	0.020604	0.008340	-0.003497	40.539839	-16.996317	0.188571
2-Chloropyridine·ICN	-7.98	0.021136	0.008417	-0.003387	39.914227	-16.022901	0.189150
3,5-Dichloropyridine·ICN	-7.06	0.022543	0.009371	-0.002607	41.564249	-11.552472	0.206460
3-Chloropyridine·ICN	-8.09	0.024187	0.010254	-0.001963	42.389226	-8.113426	0.221729
3-Acetylpyridine·ICN	-8.35	0.025203	0.010740	-0.001532	42.703262	-6.092634	0.231274
2-Isopropylpyridine·ICN	-10.10	0.025899	0.010755	-0.001718	41.710228	-6.662977	0.231049
4-Acetylpyridine·ICN	-8.42	0.024925	0.010690	-0.001643	42.847329	-6.58726	0.228895
2-Ethylpyridine·ICN	-10.10	0.026169	0.011026	-0.001530	42.020079	-5.832426	0.234878
Pyridine·ICN	-9.33	0.026362	0.011407	-0.001142	43.318434	-4.335789	0.241682
2,4,6-Trimethylpyridine·ICN	-10.43	0.024214	0.009771	-0.002127	40.349037	-8.783337	0.220684
2-Methylpyridine·ICN	-9.92	0.026462	0.011307	-0.001319	42.729898	-4.98756	0.239716
4-Methylpyridine·ICN	-9.79	0.027173	0.011879	-0.000885	43.763569	-3.259844	0.249235
4-Ethylpyridine·ICN	-9.83	0.027493	0.012069	-0.000727	43.816553	-2.637837	0.251779
3,4-Dimethylpyridine·ICN	-10.13	0.027984	0.012205	-0.000595	43.716452	-2.131862	0.256021
4-Dimethylamminopyridine·ICN	-11.47	0.030239	0.013536	0.000286	44.761469	0.946463	0.277399
2-Fluoropyridine·Br_2	-6.36	0.026469	0.011539	-0.001609	43.610668	-6.082318	0.220948
2-Chloropyridine·Br_2	-6.86	0.027828	0.012034	-0.001172	43.305803	-4.217936	0.227521
3,5-Dichloropyridine·Br_2	-6.65	0.030340	0.013592	0.000027	44.803638	0.090139	0.251893
3-Chloropyridine·Br_2	-7.70	0.034362	0.015789	0.001530	45.973996	4.454203	0.284303
3-Acetylpyridine·Br_2	-8.17	0.036279	0.016822	0.002333	46.366794	6.429726	0.299703
2-Isopropylpyridine·Br_2	-10.31	0.040023	0.018387	0.003439	45.871298	8.580079	0.323407
4-Acetylpyridine·Br_2	-8.14	0.036406	0.016875	0.002416	46.330968	6.634355	0.300611
2-Ethylpyridine·Br_2	-10.36	0.040669	0.018731	0.003771	46.032671	9.26773	0.329400
Pyridine·Br_2	-9.16	0.040032	0.018774	0.003803	46.91757	9.505097	0.328713
2,4,6-Trimethylpyridine·Br_2	-11.19	0.041869	0.019165	0.004274	45.753056	10.202562	0.342146
2-Methylpyridine·Br_2	-10.19	0.041822	0.019556	0.004177	46.829283	10.002682	0.339824
4-Methylpyridine·Br_2	-9.70	0.042166	0.019935	0.004630	47.286849	10.983018	0.345468
4-Ethylpyridine·Br_2	-9.77	0.042715	0.020245	0.004797	47.427178	11.23694	0.349407
3,4-Dimethylpyridine·Br_2	-10.15	0.044125	0.020947	0.005345	47.486287	12.117033	0.359890
4-Dimethylamminopyridine·Br_2	-11.90	0.049822	0.024079	0.007670	48.339279	15.397779	0.404448
2-Fluoropyridine·BrCN	-5.96	0.017739	0.005810	-0.004361	32.825952	-24.638191	0.145806
2-Chloropyridine·BrCN	-6.31	0.018186	0.005862	-0.004274	32.275318	-23.530323	0.145830
3,5-Dichloropyridine·BrCN	-5.41	0.018864	0.006411	-0.003745	33.976377	-19.847706	0.155438
3-Chloropyridine·BrCN	-6.15	0.019865	0.006880	-0.003300	34.603865	-16.596218	0.163897
3-Acetylpyridine·BrCN	-6.29	0.020364	0.007074	-0.003053	34.829321	-15.032446	0.168532

2-Isopropylpyridine·BrCN	-7.33	0.020907	0.007235	-0.003206	34.523539	-15.298972	0.169055
4-Acetylpyridine·BrCN	-6.37	0.020259	0.007114	-0.003108	35.067239	-15.318432	0.167675
2-Ethylpyridine·BrCN	-7.71	0.021272	0.007361	-0.003118	34.472187	-14.601552	0.171378
Pyridine·BrCN	-7.03	0.021106	0.007526	-0.002843	35.684295	-13.480876	0.174582
2,4,6-Trimethylpyridine·BrCN	-8.16	0.020581	0.006842	-0.003348	33.196131	-16.244324	0.167017
2-Methylpyridine·BrCN	-7.53	0.021361	0.007505	-0.002996	35.188224	-14.046879	0.174255
4-Methylpyridine·BrCN	-7.34	0.021523	0.007702	-0.002657	35.7911	-12.347223	0.178308
4-Ethylpyridine·BrCN	-7.35	0.021539	0.007752	-0.002647	35.927955	-12.267134	0.178448
3,4-Dimethylpyridine·BrCN	-7.55	0.021848	0.007801	-0.002582	35.782393	-11.842612	0.180825
4-Dimethylamminopyridine·BrCN	-8.42	0.023037	0.008448	-0.002082	36.673774	-9.03651	0.191643

Table S3 MP2 values of E_{BIND} (kcal/mol), $\rho_{X\cdots N}(\mathbf{r}_{bcp})$ (a.u.), absolute (SF) and percentage (SF%) halogen and nitrogen contributions (a.u.) to $\rho_{X\cdots N}(\mathbf{r}_{bcp})$ and delocalization indices between halogen and nitrogen, $\delta(\Omega_X,\Omega_N)$, in the set of complexes of substituted pyridines with I_2, ICN, Br_2 and BrCN molecules.

**Complex**	$\mathbf{E_{BIND}}$	$\boldsymbol{\rho_{X\cdots N}(\mathbf{r}_{bcp})}$	**SF(Ω_X)**	**SF(Ω_N)**	**SF%(Ω_X)**	**SF%(Ω_N)**	**$\delta(\Omega_X, \Omega_N)$**
2-Fluoropyridine·I_2	-6.32	0.024673	0.011518	-0.002036	46.661588	-8.249484	0.207526
2-Chloropyridine·I_2	-6.89	0.025138	0.011624	-0.001939	46.234365	-7.712552	0.208230
3,5-Dichloropyridine·I_2	-7.12	0.028983	0.013923	-0.000405	48.032065	-1.396989	0.241380
3-Chloropyridine·I_2	-8.10	0.032050	0.015610	0.000788	48.673113	2.456682	0.265143
3-Acetylpyridine·I_2	-8.55	0.033234	0.016261	0.001273	48.88819	3.827363	0.274286
2-Isopropylpyridine·I_2	-10.20	0.033250	0.015918	0.001041	47.851378	3.127935	0.270896
4-Acetylpyridine·I_2	-8.61	0.033344	0.016317	0.001357	48.923267	4.069025	0.275916
2-Ethylpyridine·I_2	-10.14	0.033693	0.016081	0.001274	47.77404	3.784986	0.274712
Pyridine·I_2	-9.40	0.035392	0.017420	0.002083	49.238506	5.886554	0.290937
2,4,6-Trimethylpyridine·I_2	-9.94	0.031617	0.014818	0.000598	46.890583	1.891781	0.262884
2-Methylpyridine·I_2	-9.88	0.034452	0.016720	0.001580	48.529409	4.58733	0.281917
4-Methylpyridine·I_2	-9.91	0.036706	0.018188	0.002522	49.574763	6.874263	0.301069
4-Ethylpyridine·I_2	-9.98	0.036865	0.018263	0.002583	49.566959	7.011098	0.302282
3,4-Dimethylpyridine·I_2	-10.49	0.037714	0.018745	0.002878	49.643571	7.622677	0.308946
4-Dimethylamminopyridine·I_2	-12.09	0.041363	0.020744	0.004343	50.151478	10.500389	0.337306
2-Fluoropyridine·ICN	-6.71	0.019838	0.007962	-0.003612	40.24658	-18.257218	0.168588
2-Chloropyridine·ICN	-7.11	0.020128	0.007874	-0.003499	39.309318	-17.46626	0.167965
3,5-Dichloropyridine·ICN	-6.71	0.022578	0.009418	-0.002434	41.705695	-10.780127	0.191083
3-Chloropyridine·ICN	-7.54	0.024413	0.010418	-0.001720	42.616756	-7.036648	0.205640
3-Acetylpyridine·ICN	-7.77	0.025223	0.010747	-0.001384	42.719978	-5.503216	0.212115
2-Isopropylpyridine·ICN	-8.91	0.024744	0.010366	-0.001794	41.724106	-7.220716	0.204802
4-Acetylpyridine·ICN	-7.90	0.025182	0.010842	-0.001371	42.988681	-5.4376	0.212402
2-Ethylpyridine·ICN	-9.05	0.025243	0.010622	-0.001589	41.81259	-6.253016	0.208320
Pyridine·ICN	-8.56	0.026619	0.011573	-0.000894	43.502793	-3.359495	0.222974
2,4,6-Trimethylpyridine·ICN	-8.81	0.022585	0.009025	-0.002444	39.898974	-10.803502	0.189799
2-Methylpyridine·ICN	-8.85	0.025703	0.010956	-0.001402	42.578681	-5.450538	0.213475
4-Methylpyridine·ICN	-8.94	0.027478	0.012068	-0.000634	43.957652	-2.308144	0.229688
4-Ethylpyridine·ICN	-8.98	0.027574	0.012135	-0.000553	43.933809	-2.00288	0.230407
3,4-Dimethylpyridine·ICN	-9.35	0.028162	0.012343	-0.000389	43.918253	-1.383561	0.235016

4-Dimethylamminopyridine·ICN	*-10.54*	*0.030544*	*0.013726*	*0.000518*	*44.928526*	*1.694916*	*0.254005*
2-Fluoropyridine·Br_2	*-6.01*	*0.031641*	*0.014545*	*0.000298*	*46.017212*	*0.942101*	*0.235332*
2-Chloropyridine·Br_2	*-6.64*	*0.032916*	*0.014942*	*0.000782*	*45.57245*	*2.386014*	*0.241165*
3,5-Dichloropyridine·Br_2	*-6.97*	*0.036741*	*0.017302*	*0.002405*	*47.085866*	*6.543672*	*0.270170*
3-Chloropyridine·Br_2	*-8.05*	*0.040213*	*0.019236*	*0.003755*	*47.839429*	*9.337712*	*0.293826*
3-Acetylpyridine·Br_2	*-8.56*	*0.041732*	*0.020072*	*0.004409*	*48.081142*	*10.561854*	*0.303866*
2-Isopropylpyridine·Br_2	*-10.54*	*0.043293*	*0.020541*	*0.004778*	*47.36265*	*11.017779*	*0.311179*
4-Acetylpyridine·Br_2	*-8.61*	*0.041892*	*0.020142*	*0.004547*	*48.036171*	*10.843538*	*0.305859*
2-Ethylpyridine·Br_2	*-10.48*	*0.043731*	*0.020701*	*0.005052*	*47.34882*	*11.555766*	*0.314509*
Pyridine·Br_2	*-9.48*	*0.044150*	*0.021169*	*0.005400*	*48.128788*	*12.277946*	*0.320534*
2,4,6-Trimethylpyridine·Br_2	*-10.93*	*0.044323*	*0.020874*	*0.005293*	*47.095613*	*11.941648*	*0.321013*
2-Methylpyridine·Br_2	*-10.25*	*0.044468*	*0.021337*	*0.005210*	*48.094712*	*11.744481*	*0.320552*
4-Methylpyridine·Br_2	*-10.02*	*0.045678*	*0.022184*	*0.005973*	*48.564072*	*13.07661*	*0.330659*
4-Ethylpyridine·Br_2	*-10.09*	*0.045913*	*0.022326*	*0.006041*	*48.63344*	*13.160202*	*0.332117*
3,4-Dimethylpyridine·Br_2	*-10.64*	*0.046922*	*0.022810*	*0.006418*	*48.612695*	*13.678056*	*0.339116*
4-Dimethylamminopyridine·Br_2	*-12.31*	*0.050869*	*0.025015*	*0.008058*	*49.181528*	*15.84182*	*0.365862*
2-Fluoropyridine·BrCN	*-5.19*	*0.016599*	*0.005319*	*-0.004635*	*32.174758*	*-28.035802*	*0.130578*
2-Chloropyridine·BrCN	*-5.45*	*0.016999*	*0.005335*	*-0.004495*	*31.483882*	*-26.526125*	*0.130685*
3,5-Dichloropyridine·BrCN	*-4.95*	*0.018507*	*0.006315*	*-0.003755*	*34.116198*	*-20.285674*	*0.145178*
3-Chloropyridine·BrCN	*-5.53*	*0.019929*	*0.007031*	*-0.003184*	*35.224945*	*-15.949503*	*0.155419*
3-Acetylpyridine·BrCN	*-5.65*	*0.020544*	*0.007235*	*-0.002914*	*35.325635*	*-14.226766*	*0.159873*
2-Isopropylpyridine·BrCN	*-6.54*	*0.020532*	*0.007151*	*-0.003209*	*34.690728*	*-15.567466*	*0.155992*
4-Acetylpyridine·BrCN	*-5.77*	*0.020477*	*0.007320*	*-0.002922*	*35.677689*	*-14.241607*	*0.159902*
2-Ethylpyridine·BrCN	*-6.64*	*0.020930*	*0.007366*	*-0.003062*	*35.099654*	*-14.589477*	*0.158597*
Pyridine·BrCN	*-6.22*	*0.021611*	*0.007884*	*-0.002580*	*36.49672*	*-11.942382*	*0.167400*
2,4,6-Trimethylpyridine·BrCN	*-6.67*	*0.019401*	*0.006411*	*-0.003552*	*33.063941*	*-18.321583*	*0.148493*
2-Methylpyridine·BrCN	*-6.49*	*0.021242*	*0.007567*	*-0.002909*	*35.669793*	*-13.711565*	*0.162286*
4-Methylpyridine·BrCN	*-6.48*	*0.022221*	*0.008170*	*-0.002337*	*36.736796*	*-10.510204*	*0.171789*
4-Ethylpyridine·BrCN	*-6.50*	*0.022287*	*0.008240*	*-0.002314*	*36.879755*	*-10.358274*	*0.172252*
3,4-Dimethylpyridine·BrCN	*-6.75*	*0.022710*	*0.008253*	*-0.002201*	*36.585212*	*-9.757*	*0.175246*
4-Dimethylamminopyridine·BrCN	*-7.50*	*0.024379*	*0.009284*	*-0.001547*	*38.034579*	*-6.335752*	*0.187552*

Table S4 M11 values of E_{BIND} (kcal/mol), $\rho_{X\cdots N}(\mathbf{r}_{bcp})$ (a.u.), absolute (SF) and percentage (SF%) halogen and nitrogen contributions (a.u.) to $\rho_{X\cdots N}(\mathbf{r}_{bcp})$ and delocalization indices between halogen and nitrogen, $\delta(\Omega_X,\Omega_N)$, in the set of complexes of substituted pyridines with I_2, ICN, Br_2 and BrCN molecules.

**Complex**	E_{BIND}	$\rho_{X\cdots N}(\mathbf{r}_{bcp})$	**SF(Ω_X)**	**SF(Ω_N)**	**SF%(Ω_X)**	**SF%(Ω_N)**	**$\delta(\Omega_X, \Omega_N)$**
2-Fluoropyridine·I_2	*-4.91*	*0.021184*	*0.009469*	*-0.003378*	*44.627875*	*-15.919997*	*0.191049*
2-Chloropyridine·I_2	*-5.47*	*0.022318*	*0.009936*	*-0.003109*	*44.524428*	*-13.93399*	*0.197438*
3,5-Dichloropyridine·I_2	*-4.94*	*0.023202*	*0.010606*	*-0.002497*	*45.687019*	*-10.758134*	*0.211039*
3-Chloropyridine·I_2	*-5.78*	*0.025384*	*0.011748*	*-0.001664*	*46.279071*	*-6.555755*	*0.232177*
3-Acetylpyridine·I_2	*-6.09*	*0.026509*	*0.012365*	*-0.001210*	*46.592281*	*-4.559379*	*0.242894*
2-Isopropylpyridine·I_2	*-7.95*	*0.027847*	*0.012670*	*-0.001137*	*45.46303*	*-4.081224*	*0.250378*
4-Acetylpyridine·I_2	*-6.10*	*0.026465*	*0.012361*	*-0.001229*	*46.663373*	*-4.638278*	*0.242745*
2-Ethylpyridine·I_2	*-7.98*	*0.028458*	*0.013065*	*-0.000842*	*45.919487*	*-2.959066*	*0.256761*

Pyridine·I_2	-6.89	0.028771	0.013578	-0.000426	47.205374	-1.480326	0.264061
2,4,6-Trimethylpyridine·I_2	-8.37	0.027361	0.012276	-0.001140	44.852661	-4.164629	0.251280
2-Methylpyridine·I_2	-7.71	0.029336	0.013707	-0.000456	46.703973	-1.553028	0.266737
4-Methylpyridine·I_2	-7.29	0.030076	0.014323	0.000000	47.641146	0.000548	0.276475
4-Ethylpyridine·I_2	-7.33	0.030415	0.014497	0.000132	47.672534	0.433551	0.279348
3,4-Dimethylpyridine·I_2	-7.58	0.031291	0.014983	0.000422	47.832282	1.345914	0.287043
4-Dimethylamminopyridine·I_2	-8.91	0.035405	0.017206	0.002022	48.60731	5.711869	0.326071
2-Fluoropyridine·ICN	-6.41	0.018996	0.007455	-0.004005	39.269195	-21.09487	0.168764
2-Chloropyridine·ICN	-6.88	0.019868	0.007665	-0.003784	38.785066	-19.148445	0.172762
3,5-Dichloropyridine·ICN	-5.84	0.020814	0.008446	-0.003136	40.557777	-15.060589	0.186334
3-Chloropyridine·ICN	-6.79	0.022092	0.009127	-0.002648	41.310597	-11.987145	0.198665
3-Acetylpyridine·ICN	-6.98	0.022929	0.009380	-0.002284	41.180915	-10.026988	0.207087
2-Isopropylpyridine·ICN	-8.25	0.023330	0.009575	-0.002443	40.984978	-10.45853	0.207344
4-Acetylpyridine·ICN	-7.07	0.022603	0.009363	-0.002414	41.453027	-10.688814	0.203823
2-Ethylpyridine·ICN	-8.81	0.023889	0.009787	-0.002337	40.793112	-9.739792	0.211415
Pyridine·ICN	-7.91	0.023830	0.010031	-0.001993	42.108414	-8.365603	0.215659
2,4,6-Trimethylpyridine·ICN	-9.23	0.022732	0.008997	-0.002633	39.557428	-11.575636	0.203692
2-Methylpyridine·ICN	-8.56	0.024207	0.010093	-0.002104	41.723132	-8.696023	0.216504
4-Methylpyridine·ICN	-8.32	0.024436	0.010384	-0.001812	42.5028	-7.416304	0.221722
4-Ethylpyridine·ICN	-8.35	0.024604	0.010488	-0.001707	42.542115	-6.925272	0.223073
3,4-Dimethylpyridine·ICN	-8.60	0.024956	0.010542	-0.001625	42.372078	-6.532802	0.226222
4-Dimethylamminopyridine·ICN	-9.80	0.026738	0.011607	-0.000937	43.402415	-3.503963	0.244561
2-Fluoropyridine·Br_2	-5.28	0.024492	0.010464	-0.002325	42.735683	-9.495922	0.202198
2-Chloropyridine·Br_2	-5.89	0.026124	0.011056	-0.001797	42.478973	-6.903322	0.211634
3,5-Dichloropyridine·Br_2	-5.54	0.027446	0.012028	-0.000996	43.813219	-3.629239	0.227620
3-Chloropyridine·Br_2	-6.43	0.030173	0.013515	0.000006	44.768811	0.01967	0.251113
3-Acetylpyridine·Br_2	-6.80	0.031534	0.014240	0.000572	45.167432	1.814417	0.263186
2-Isopropylpyridine·Br_2	-8.90	0.034491	0.015385	0.001293	44.49162	3.738866	0.282826
4-Acetylpyridine·Br_2	-6.79	0.031531	0.014235	0.000611	45.084656	1.935924	0.262961
2-Ethylpyridine·Br_2	-8.92	0.034787	0.015573	0.001511	44.671698	4.333099	0.286149
Pyridine·Br_2	-7.63	0.034115	0.015554	0.001568	47.205374	4.597421	0.284787
2,4,6-Trimethylpyridine·Br_2	-9.68	0.035914	0.015953	0.001963	44.368449	5.458835	0.298572
2-Methylpyridine·Br_2	-8.63	0.035549	0.016150	0.001760	45.52634	4.961141	0.294163
4-Methylpyridine·Br_2	-8.06	0.035630	0.016370	0.002147	45.924215	6.023922	0.297723
4-Ethylpyridine·Br_2	-8.10	0.035893	0.016554	0.002203	46.090301	6.13256	0.299708
3,4-Dimethylpyridine·Br_2	-8.37	0.036817	0.016928	0.002546	46.049308	6.92495	0.307254
4-Dimethylamminopyridine·Br_2	-9.81	0.041720	0.019613	0.004531	47.009049	10.859771	0.348729
2-Fluoropyridine·BrCN	-5.33	0.016535	0.005166	-0.004709	31.303047	-28.535174	0.132493
2-Chloropyridine·BrCN	-5.76	0.017306	0.005404	-0.004527	31.343837	-26.258242	0.135565
3,5-Dichloropyridine·BrCN	-4.74	0.017994	0.005993	-0.003994	33.283603	-22.183642	0.145484
3-Chloropyridine·BrCN	-5.49	0.018917	0.006426	-0.003613	33.915689	-19.065619	0.152979
3-Acetylpyridine·BrCN	-5.57	0.019329	0.006540	-0.003369	33.952093	-17.488846	0.157544
2-Isopropylpyridine·BrCN	-6.65	0.020274	0.006940	-0.003403	34.162219	-16.75255	0.161296
4-Acetylpyridine·BrCN	-5.69	0.019189	0.006571	-0.003467	34.138493	-18.014434	0.155367
2-Ethylpyridine·BrCN	-7.12	0.020517	0.006975	-0.003388	33.859142	-16.445468	0.162564
Pyridine·BrCN	-6.32	0.020095	0.007029	-0.003159	34.987733	-15.726915	0.163829
2,4,6-Trimethylpyridine·BrCN	-7.63	0.020210	0.006681	-0.003458	32.990038	-17.072106	0.161170
2-Methylpyridine·BrCN	-6.87	0.020544	0.007109	-0.003262	34.684955	-15.917928	0.165049
4-Methylpyridine·BrCN	-6.63	0.020487	0.007192	-0.002985	35.081859	-14.560588	0.167438

4-Ethylpyridine·BrCN	-6.65	0.020536	0.007255	-0.002960	35.256562	-14.38154	0.167785
3,4-Dimethylpyridine·BrCN	-6.83	0.020599	0.007246	-0.003063	35.159118	-14.864444	0.167279
4-Dimethylamminopyridine·BrCN	-7.69	0.021897	0.007880	-0.002441	35.976189	-11.1461	0.180320

Table S5 ωB97X values of E_{BIND} (kcal/mol), $\rho_{X\cdots N}(\mathbf{r}_{bcp})$ (a.u.), absolute (SF) and percentage (SF%) halogen and nitrogen contributions (a.u.) to $\rho_{X\cdots N}(\mathbf{r}_{bcp})$ and delocalization indices between halogen and nitrogen, $\delta(\Omega_X,\Omega_N)$, in the set of complexes of substituted pyridines with I_2, ICN, Br_2 and BrCN molecules.

Complex	E_{BIND}	$\rho_{X\cdots N}(\mathbf{r}_{bcp})$	SF(Ω_X)	SF(Ω_N)	SF%(Ω_X)	SF%(Ω_N)	$\delta(\Omega_X, \Omega_N)$
2-Fluoropyridine·I_2	-6.24	0.021571	0.009686	-0.003188	44.896892	-14.776846	0.206764
2-Chloropyridine·I_2	-6.72	0.022192	0.009886	-0.003050	44.501781	-13.727281	0.208350
3,5-Dichloropyridine·I_2	-6.39	0.024066	0.011087	-0.002157	46.058855	-8.958811	0.230826
3-Chloropyridine·I_2	-7.29	0.026105	0.012182	-0.001348	46.618127	-5.15921	0.250383
3-Acetylpyridine·I_2	-7.64	0.027196	0.012773	-0.000897	46.882821	-3.293787	0.260772
2-Isopropylpyridine·I_2	-8.92	0.027092	0.012329	-0.001258	45.513138	-4.645595	0.253933
4-Acetylpyridine·I_2	-7.62	0.027069	0.012718	-0.000942	46.907899	-3.476011	0.259856
2-Ethylpyridine·I_2	-8.99	0.027522	0.012547	-0.001019	45.665816	-3.709249	0.259107
Pyridine·I_2	-8.43	0.029194	0.013827	-0.000202	47.377445	-0.692074	0.278549
2,4,6-Trimethylpyridine·I_2	-9.06	0.025413	0.011269	-0.001685	44.36017	-6.631444	0.244308
2-Methylpyridine·I_2	-8.87	0.028310	0.013174	-0.000699	46.52497	-2.467565	0.268396
4-Methylpyridine·I_2	-8.86	0.030434	0.014538	0.000206	47.799122	0.678225	0.290025
4-Ethylpyridine·I_2	-8.88	0.030586	0.014607	0.000262	47.786995	0.857444	0.291329
3,4-Dimethylpyridine·I_2	-9.16	0.031360	0.015044	0.000514	47.907374	1.635526	0.297974
4-Dimethylamminopyridine·I_2	-10.47	0.034813	0.016898	0.001889	48.53666	5.427082	0.329800
2-Fluoropyridine·ICN	-7.84	0.020436	0.008214	-0.003550	40.314376	-17.425283	0.194166
2-Chloropyridine·ICN	-8.28	0.020858	0.008185	-0.003391	39.441858	-16.339059	0.193901
3,5-Dichloropyridine·ICN	-7.44	0.022441	0.009287	-0.002568	41.368778	-11.437785	0.213555
3-Chloropyridine·ICN	-8.46	0.023776	0.010009	-0.002022	42.050437	-8.493724	0.226526
3-Acetylpyridine·ICN	-8.71	0.024443	0.010248	-0.001718	42.067577	-7.052237	0.233347
2-Isopropylpyridine·ICN	-10.09	0.024339	0.009802	-0.002137	40.612761	-8.852584	0.226079
4-Acetylpyridine·ICN	-8.79	0.024333	0.010330	-0.001777	42.396095	-7.294187	0.232273
2-Ethylpyridine·ICN	-10.15	0.024574	0.010160	-0.001952	41.307477	-7.935398	0.229507
Pyridine·ICN	-9.67	0.025468	0.010886	-0.001378	42.771621	-5.412183	0.242695
2,4,6-Trimethylpyridine·ICN	-10.28	0.022570	0.008904	-0.002564	39.479316	-11.367623	0.214349
2-Methylpyridine·ICN	-10.05	0.024974	0.010481	-0.001743	41.937177	-6.973486	0.235408
4-Methylpyridine·ICN	-10.12	0.026144	0.011279	-0.001169	43.183282	-4.475204	0.249283
4-Ethylpyridine·ICN	-10.15	0.026202	0.011324	-0.001100	43.135719	-4.191776	0.249777
3,4-Dimethylpyridine·ICN	-10.43	0.026620	0.011434	-0.001004	43.042316	-3.77875	0.253415
4-Dimethylamminopyridine·ICN	-11.71	0.028646	0.012616	-0.000208	44.028572	-0.726807	0.272922
2-Fluoropyridine·Br_2	-6.40	0.024297	0.010411	-0.002401	42.983332	-9.913154	0.211053
2-Chloropyridine·Br_2	-6.87	0.025443	0.010787	-0.001984	42.501003	-7.818174	0.216456
3,5-Dichloropyridine·Br_2	-6.71	0.027651	0.012197	-0.000918	44.102722	-3.320978	0.239102
3-Chloropyridine·Br_2	-7.61	0.030074	0.013533	-0.000032	45.018219	-0.107212	0.260141
3-Acetylpyridine·Br_2	-8.00	0.031306	0.014191	0.000502	45.323954	1.60227	0.270693
2-Isopropylpyridine·Br_2	-9.42	0.032427	0.014411	0.000612	44.299455	1.882807	0.274839

4-Acetylpyridine·Br_2	-7.97	0.031236	0.014147	0.000508	45.250173	1.624067	0.270115
2-Ethylpyridine·Br_2	-9.49	0.032916	0.014658	0.000885	44.409563	2.682581	0.279714
Pyridine·Br_2	-8.79	0.033582	0.015355	0.001374	45.740373	4.092423	0.289316
2,4,6-Trimethylpyridine·Br_2	-9.88	0.032157	0.014084	0.000638	43.749053	1.98032	0.277300
2-Methylpyridine·Br_2	-9.38	0.033683	0.015257	0.001099	45.50058	3.277111	0.287756
4-Methylpyridine·Br_2	-9.21	0.034823	0.016030	0.001851	46.02253	5.314181	0.299879
4-Ethylpyridine·Br_2	-9.24	0.034939	0.016104	0.001862	46.107332	5.331978	0.300853
3,4-Dimethylpyridine·Br_2	-9.51	0.035667	0.016435	0.002119	46.088365	5.942436	0.306710
4-Dimethylamminopyridine·Br_2	-10.80	0.039246	0.018396	0.003579	46.867891	9.118778	0.337152
2-Fluoropyridine·BrCN	-6.38	0.017602	0.005747	-0.004419	32.711676	-25.152816	0.151160
2-Chloropyridine·BrCN	-6.74	0.017936	0.005719	-0.004317	31.985334	-24.142976	0.150523
3,5-Dichloropyridine·BrCN	-5.91	0.018863	0.006421	-0.003720	34.020629	-19.711118	0.162711
3-Chloropyridine·BrCN	-6.69	0.019841	0.006893	-0.003285	34.684473	-16.531765	0.171419
3-Acetylpyridine·BrCN	-6.83	0.020307	0.007033	-0.003058	34.752585	-15.112945	0.175969
2-Isopropylpyridine·BrCN	-7.75	0.020441	0.007009	-0.003331	34.108609	-16.207204	0.172989
4-Acetylpyridine·BrCN	-6.92	0.020225	0.007105	-0.003102	35.077539	-15.315282	0.175186
2-Ethylpyridine·BrCN	-8.04	0.020806	0.007161	-0.003224	34.318247	-15.44977	0.175359
Pyridine·BrCN	-7.58	0.021003	0.007489	-0.002865	35.66711	-13.646017	0.181878
2,4,6-Trimethylpyridine·BrCN	-8.38	0.019747	0.006458	-0.003558	32.620556	-17.970072	0.168076
2-Methylpyridine·BrCN	-7.94	0.020975	0.007334	-0.003090	35.040082	-14.764021	0.179039
4-Methylpyridine·BrCN	-7.91	0.021422	0.007673	-0.002677	35.797662	-12.489082	0.185800
4-Ethylpyridine·BrCN	-7.93	0.021463	0.007728	-0.002661	35.944149	-12.376452	0.186124
3,4-Dimethylpyridine·BrCN	-8.12	0.021713	0.007634	-0.002611	35.390956	-12.106062	0.188116
4-Dimethylamminopyridine·BrCN	-9.01	0.022823	0.008370	-0.002140	36.667067	-9.375384	0.198793

Table S6 ωB97XD values of E_{BIND} (kcal/mol), $\rho_{X\cdots N}(\mathbf{r}_{bcp})$ (a.u.), absolute (SF) and percentage (SF%) halogen and nitrogen contributions (a.u.) to $\rho_{X\cdots N}(\mathbf{r}_{bcp})$ and delocalization indices between halogen and nitrogen, $\delta(\Omega_X,\Omega_N)$, in the set of complexes of substituted pyridines with I_2, ICN, Br_2 and BrCN molecules.

**Complex**	E_{BIND}	$\rho_{X\cdots N}(\mathbf{r}_{bcp})$	**SF(Ω_X)**	**SF(Ω_N)**	**SF%(Ω_X)**	**SF%(Ω_N)**	**$\delta(\Omega_X, \Omega_N)$**
2-Fluoropyridine·I_2	-6.16	0.022467	0.010209	-0.002852	45.418752	-12.68573	0.214547
2-Chloropyridine·I_2	-6.47	0.022789	0.010247	-0.002833	44.925315	-12.418917	0.213795
3,5-Dichloropyridine·I_2	-6.41	0.025114	0.011693	-0.001789	46.560551	-7.121946	0.239791
3-Chloropyridine·I_2	-7.35	0.027556	0.013015	-0.000834	47.181014	-3.021717	0.263137
3-Acetylpyridine·I_2	-7.80	0.029004	0.013798	-0.000255	47.50648	-0.877481	0.276402
2-Isopropylpyridine·I_2	-9.47	0.028975	0.013417	-0.000564	46.258987	-1.943097	0.272321
4-Acetylpyridine·I_2	-7.70	0.028758	0.013667	-0.000345	47.496747	-1.198261	0.274542
2-Ethylpyridine·I_2	-9.46	0.029444	0.013609	-0.000339	46.361547	-1.153659	0.277545
Pyridine·I_2	-8.56	0.031053	0.014886	0.000472	47.953934	1.52194	0.295025
2,4,6-Trimethylpyridine·I_2	-9.49	0.026841	0.012085	-0.001158	45.075196	-4.320578	0.259401
2-Methylpyridine·I_2	-9.18	0.030102	0.014193	-0.000050	47.160957	-0.166031	0.285193
4-Methylpyridine·I_2	-9.06	0.032553	0.015742	0.000973	48.394851	2.991202	0.308588
4-Ethylpyridine·I_2	-9.12	0.032755	0.015839	0.001055	48.377108	3.22363	0.310268
3,4-Dimethylpyridine·I_2	-9.46	0.033682	0.016360	0.001362	48.507204	4.038522	0.318156
4-Dimethylamminopyridine·I_2	-10.90	0.037464	0.018404	0.002871	49.114366	7.661779	0.352470

2-Fluoropyridine·ICN	-7.59	0.020607	0.008308	-0.003454	40.414289	-16.800919	0.194820
2-Chloropyridine·ICN	-7.84	0.020829	0.008167	-0.003373	39.440234	-16.290236	0.193139
3,5-Dichloropyridine·ICN	-7.24	0.022918	0.009540	-0.002389	41.621563	-10.422997	0.216505
3-Chloropyridine·ICN	-8.26	0.024203	0.010244	-0.001850	42.256289	-7.630853	0.229530
3-Acetylpyridine·ICN	-8.57	0.024904	0.010441	-0.001535	42.161643	-6.199522	0.236703
2-Isopropylpyridine·ICN	-10.25	0.024902	0.010175	-0.001904	41.117573	-7.693386	0.231922
4-Acetylpyridine·ICN	-8.59	0.024747	0.010551	-0.001604	42.576477	-6.473394	0.235355
2-Ethylpyridine·ICN	-10.22	0.025087	0.010445	-0.001738	41.652836	-6.932392	0.234737
Pyridine·ICN	-9.47	0.025855	0.011096	-0.001210	42.945986	-4.683899	0.245844
2,4,6-Trimethylpyridine·ICN	-10.31	0.023077	0.009189	-0.002356	39.849509	-10.218813	0.219643
2-Methylpyridine·ICN	-9.98	0.025373	0.010702	-0.001571	42.137441	-6.186706	0.239284
4-Methylpyridine·ICN	-9.96	0.026620	0.011535	-0.000973	43.377168	-3.658301	0.253205
4-Ethylpyridine·ICN	-10.01	0.026716	0.011602	-0.000890	43.350292	-3.326987	0.254015
3,4-Dimethylpyridine·ICN	-10.33	0.027228	0.011757	-0.000762	43.273082	-2.802847	0.258488
4-Dimethylamminopyridine·ICN	-11.65	0.029486	0.013071	0.000115	44.318143	0.391085	0.279955
2-Fluoropyridine·Br_2	-6.30	0.026182	0.011450	-0.001711	43.773828	-6.539641	0.226375
2-Chloropyridine·Br_2	-6.62	0.027061	0.011672	-0.001396	43.246499	-5.17161	0.229869
3,5-Dichloropyridine·Br_2	-6.77	0.030212	0.013599	-0.000009	45.009014	-0.028402	0.259125
3-Chloropyridine·Br_2	-7.80	0.033512	0.015410	0.001213	45.998127	3.621021	0.286785
3-Acetylpyridine·Br_2	-8.30	0.034990	0.016206	0.001859	46.293507	5.309364	0.299068
2-Isopropylpyridine·Br_2	-10.12	0.036303	0.016534	0.002073	45.428142	5.695654	0.306705
4-Acetylpyridine·Br_2	-8.21	0.034834	0.016116	0.001825	46.234446	5.235382	0.298101
2-Ethylpyridine·Br_2	-10.12	0.036633	0.016680	0.002280	45.53356	6.223526	0.310175
Pyridine·Br_2	-9.12	0.036882	0.017013	0.002628	46.315798	7.154063	0.315447
2,4,6-Trimethylpyridine·Br_2	-10.57	0.036543	0.016460	0.002276	45.023114	6.225775	0.313434
2-Methylpyridine·Br_2	-9.89	0.037145	0.017157	0.002388	46.309995	6.446053	0.315947
4-Methylpyridine·Br_2	-9.64	0.038247	0.017920	0.003131	46.843453	8.185591	0.327013
4-Ethylpyridine·Br_2	-9.69	0.038448	0.018040	0.003184	46.930038	8.282334	0.328556
3,4-Dimethylpyridine·Br_2	-10.05	0.039474	0.018526	0.003553	46.938241	9.002207	0.336482
4-Dimethylamminopyridine·Br_2	-11.55	0.043944	0.020985	0.005369	47.746502	12.216004	0.372727
2-Fluoropyridine·BrCN	-6.06	0.017531	0.005727	-0.004409	32.763614	-25.224592	0.149545
2-Chloropyridine·BrCN	-6.25	0.017759	0.005666	-0.004351	31.983909	-24.557716	0.148337
3,5-Dichloropyridine·BrCN	-5.62	0.019187	0.006598	-0.003604	34.377043	-18.77847	0.163760
3-Chloropyridine·BrCN	-6.39	0.020287	0.007149	-0.003131	35.181679	-15.408355	0.173710
3-Acetylpyridine·BrCN	-6.58	0.020833	0.007325	-0.002866	35.258835	-13.798171	0.178757
2-Isopropylpyridine·BrCN	-7.67	0.021053	0.007335	-0.003100	34.707722	-14.667461	0.177383
4-Acetylpyridine·BrCN	-6.62	0.020698	0.007367	-0.002932	35.53256	-14.143062	0.177719
2-Ethylpyridine·BrCN	-7.96	0.021399	0.007496	-0.002987	34.979095	-13.939397	0.179869
Pyridine·BrCN	-7.29	0.021555	0.007786	-0.002658	36.135865	-12.335089	0.185117
2,4,6-Trimethylpyridine·BrCN	-8.25	0.020132	0.006683	-0.003398	33.137555	-16.850362	0.171283
2-Methylpyridine·BrCN	-7.76	0.021527	0.007635	-0.002885	35.509589	-13.417904	0.182835
4-Methylpyridine·BrCN	-7.65	0.022049	0.008011	-0.002443	36.299258	-11.070498	0.189618
4-Ethylpyridine·BrCN	-7.68	0.022111	0.008076	-0.002421	36.472699	-10.935956	0.190102
3,4-Dimethylpyridine·BrCN	-7.91	0.022415	0.008009	-0.002354	35.963045	-10.570602	0.192553
4-Dimethylamminopyridine·BrCN	-8.82	0.023569	0.008763	-0.001867	37.168654	-7.918775	0.203702

Table S7 Ranking of substituted pyridines according to the experimental value of pKBI2=-log10[Kc], being Kc the equilibrium constant for the reaction Py + I2→ Py···I-I in hexane at 298 K

Substituted pyridine	pK_{BI2}
2-Fluoropyridine	0.43
2-Chloropyridine	0.70
3,5-Dichloropyridine	0.81
3-Chloropyridine	1.38
3-Acetylpyridine	1.52
2-Isopropylpyridine	1.65
4-Acetylpyridine	1.80
2-Ethylpyridine	2.09
Pyridine	2.22
2,4,6-Trimethylpyridine	2.28
2-Methylpyridine	2.35
4-Methylpyridine	2.57
4-Ethylpyridine	2.61
3,4-Dimethylpyridine	2.84
4-Dimethylamminopyridine	3.78

Table S8 IQA binding energies (a.u.) and their respective contributions (a.u.) computed at B3LYP level of theory for the set of complexes of substituted pyridines with I_2, ICN, Br_2 and BrCN molecules.

Complex	E_{IQA}^{BIND}	$E_{IQA}^{Inter(F1\cdots F2)}$	$\Delta E_{IQA}^{Intra(F1)}$	$\Delta E_{IQA}^{Intra(F2)}$	$\Delta E_{IQA}^{Inter(F1)}$	$\Delta E_{IQA}^{Inter(F2)}$
2-Fluoropyridine·I_2	-0.008185	-0.063621	0.010234	0.059935	0.002457	-0.01719
2-Chloropyridine·I_2	-0.008486	-0.068161	0.012537	0.060274	0.002741	-0.015877
3,5-Dichloropyridine·I_2	-0.009816	-0.072353	0.010326	0.062999	0.003074	-0.013862
3-Chloropyridine·I_2	-0.011568	-0.081254	0.01045	0.068334	0.003762	-0.012861
3-Acetylpyridine·I_2	-0.011913	-0.084909	0.010349	0.07029	0.004147	-0.011789
2-Isopropylpyridine·I_2	-0.0123	-0.10802	0.017734	0.080054	0.004653	-0.006721
4-Acetylpyridine·I_2	-0.012252	-0.084989	0.010814	0.067586	0.003933	-0.009596
2-Ethylpyridine·I_2	-0.013162	-0.097897	0.014009	0.074258	0.004712	-0.008244
Pyridine·I_2	-0.013961	-0.090967	0.010493	0.073385	0.004508	-0.011381
2,4,6-Trimethylpyridine·I_2	-0.012929	-0.103224	0.015034	0.076428	0.00496	-0.006128

2-Methylpyridine·I_2	-0.014034	-0.097117	0.012266	0.075598	0.004741	-0.009522
4-Methylpyridine·I_2	-0.01455	-0.094786	0.010534	0.076527	0.004773	-0.011599
4-Ethylpyridine·I_2	-0.015422	-0.095212	0.010522	0.076365	0.004798	-0.011895
3,4-Dimethylpyridine·I_2	-0.015629	-0.097719	0.010495	0.076757	0.005012	-0.010174
4-Dimethylamminopyridine·I_2	-0.018572	-0.108282	0.011038	0.08834	0.005608	-0.015276
2-Fluoropyridine·ICN	-0.009352	-0.052001	0.034595	0.050352	-0.020604	-0.021694
2-Chloropyridine·ICN	-0.009288	-0.055365	0.036512	0.050273	-0.020161	-0.020547
3,5-Dichloropyridine·ICN	-0.009312	-0.0561	0.032997	0.052617	-0.017814	-0.021012
3-Chloropyridine·ICN	-0.01074	-0.062009	0.037055	0.05514	-0.020735	-0.020191
3-Acetylpyridine·ICN	-0.010981	-0.064438	0.037566	0.055941	-0.020765	-0.019285
2-Isopropylpyridine·ICN	-0.010619	-0.070749	0.044106	0.055398	-0.021585	-0.01779
4-Acetylpyridine·ICN	-0.011221	-0.064341	0.038341	0.053546	-0.021526	-0.017241
2-Ethylpyridine·ICN	-0.011091	-0.073856	0.043949	0.058456	-0.023161	-0.016479
Pyridine·ICN	-0.012848	-0.069037	0.041726	0.057846	-0.024047	-0.019335
2,4,6-Trimethylpyridine·ICN	-0.012028	-0.076204	0.04519	0.05778	-0.023418	-0.015376
2-Methylpyridine·ICN	-0.012725	-0.073027	0.043003	0.058841	-0.023588	-0.017954
4-Methylpyridine·ICN	-0.013252	-0.071859	0.043697	0.05985	-0.02551	-0.01943
4-Ethylpyridine·ICN	-0.013885	-0.072148	0.043887	0.059659	-0.025631	-0.019653
3,4-Dimethylpyridine·ICN	-0.014385	-0.074037	0.045145	0.059867	-0.026529	-0.018831
4-Dimethylamminopyridine·ICN	-0.016415	-0.081839	0.050543	0.067278	-0.030345	-0.022052
2-Fluoropyridine·Br_2	-0.010483	-0.07414	0.00252	0.067815	0.00451	-0.011189
2-Chloropyridine·Br_2	-0.011018	-0.0804	0.004241	0.069346	0.005119	-0.009323
3,5-Dichloropyridine·Br_2	-0.0124	-0.084063	0.000567	0.069703	0.006058	-0.004665
3-Chloropyridine·Br_2	-0.014592	-0.092823	-0.001067	0.074161	0.007182	-0.002046
3-Acetylpyridine·Br_2	-0.015642	-0.097193	-0.001866	0.076046	0.007631	-0.000259
2-Isopropylpyridine·Br_2	-0.017924	-0.11376	0.00038	0.081519	0.009069	0.004867
4-Acetylpyridine·Br_2	-0.015327	-0.097009	-0.001387	0.0733	0.007593	0.002175
2-Ethylpyridine·Br_2	-0.01742	-0.11366	0.000168	0.081991	0.009088	0.004994
Pyridine·Br_2	-0.017288	-0.102629	-0.003031	0.078529	0.008581	0.001262
2,4,6-Trimethylpyridine·Br_2	-0.017935	-0.124355	0.000883	0.08913	0.010076	0.006331
2-Methylpyridine·Br_2	-0.018065	-0.111466	-0.001725	0.082691	0.009176	0.003259
4-Methylpyridine·Br_2	-0.01841	-0.106312	-0.003756	0.081327	0.008992	0.001339
4-Ethylpyridine·Br_2	-0.018618	-0.106839	-0.00385	0.081517	0.009061	0.001493
3,4-Dimethylpyridine·Br_2	-0.019559	-0.109254	-0.004396	0.08112	0.009437	0.003534
4-Dimethylamminopyridine·Br_2	-0.022297	-0.118769	-0.006034	0.092526	0.01063	-0.000649
2-Fluoropyridine·BrCN	-0.007672	-0.039641	0.02986	0.037988	-0.01918	-0.0167
2-Chloropyridine·BrCN	-0.007717	-0.042349	0.031466	0.038007	-0.018969	-0.015873
3,5-Dichloropyridine·BrCN	-0.00725	-0.041793	0.027567	0.039291	-0.016146	-0.016168
3-Chloropyridine·BrCN	-0.008525	-0.046074	0.031372	0.040652	-0.019154	-0.015322
3-Acetylpyridine·BrCN	-0.008604	-0.047827	0.031667	0.041058	-0.019132	-0.01437
2-Isopropylpyridine·BrCN	-0.008426	-0.05423	0.036679	0.042921	-0.021533	-0.012262
4-Acetylpyridine·BrCN	-0.008678	-0.047662	0.032366	0.039327	-0.019839	-0.01287
2-Ethylpyridine·BrCN	-0.009364	-0.055352	0.037152	0.042939	-0.021628	-0.012475
Pyridine·BrCN	-0.010003	-0.051121	0.035724	0.042237	-0.022509	-0.014334
2,4,6-Trimethylpyridine·BrCN	-0.009206	-0.057941	0.038936	0.043637	-0.022253	-0.011585
2-Methylpyridine·BrCN	-0.009671	-0.054123	0.036704	0.04322	-0.022184	-0.013289
4-Methylpyridine·BrCN	-0.010606	-0.053018	0.037412	0.04329	-0.023872	-0.014418
4-Ethylpyridine·BrCN	-0.010425	-0.05319	0.037529	0.043466	-0.023977	-0.014254
3,4-Dimethylpyridine·BrCN	-0.01121	-0.05443	0.038643	0.043259	-0.02487	-0.013812

4-Dimethylamminopyridine·BrCN	*-0.012248*	*-0.059582*	*0.043294*	*0.048018*	*-0.028508*	*-0.01547*

Table S9 B3LYP values, in kcal/mol, of IQA binding energy, E_{IQA}^{BIND}, exact binding energy, E_{BIND}, and their difference, together with relative percentage error, computed for the set of complexes of substituted pyridines with I_2, ICN, Br_2 and BrCN molecules.

Complex	E_{IQA}^{BIND}	E_{BIND}	$E_{IQA}^{BIND} - E_{BIND}$	$(E_{IQA}^{BIND} - E_{BIND})/E_{BIND}$
2-Fluoropyridine·I_2	-5.14	-4.72	-0.42	8.90
2-Chloropyridine·I_2	-5.33	-4.69	-0.64	13.65
3,5-Dichloropyridine·I_2	-6.16	-5.28	-0.88	16.67
3-Chloropyridine·I_2	-7.26	-6.49	-0.77	11.86
3-Acetylpyridine·I_2	-7.48	-6.96	-0.52	7.47
2-Isopropylpyridine·I_2	-7.72	-7.55	-0.17	2.25
4-Acetylpyridine·I_2	-7.69	-6.94	-0.75	10.81
2-Ethylpyridine·I_2	-8.26	-7.63	-0.63	8.26
Pyridine·I_2	-8.76	-8.01	-0.75	9.36
2,4,6-Trimethylpyridine·I_2	-8.11	-7.33	-0.78	10.64
2-Methylpyridine·I_2	-8.81	-7.87	-0.94	11.94
4-Methylpyridine·I_2	-9.13	-8.60	-0.53	6.16
4-Ethylpyridine·I_2	-9.68	-8.65	-1.03	11.91
3,4-Dimethylpyridine·I_2	-9.81	-9.05	-0.76	8.40
4-Dimethylamminopyridine·I_2	-11.65	-10.81	-0.84	7.77
2-Fluoropyridine·ICN	-5.87	-5.58	-0.29	5.20
2-Chloropyridine·ICN	-5.83	-5.46	-0.37	6.78
3,5-Dichloropyridine·ICN	-5.84	-5.34	-0.50	9.36
3-Chloropyridine·ICN	-6.74	-6.41	-0.33	5.15
3-Acetylpyridine·ICN	-6.89	-6.65	-0.24	3.61
2-Isopropylpyridine·ICN	-6.66	-7.11	0.45	-6.33
4-Acetylpyridine·ICN	-7.04	-6.74	-0.30	4.45
2-Ethylpyridine·ICN	-6.96	-7.18	0.22	-3.06
Pyridine·ICN	-8.06	-7.69	-0.37	4.81
2,4,6-Trimethylpyridine·ICN	-7.55	-6.79	-0.76	11.19
2-Methylpyridine·ICN	-7.99	-7.42	-0.57	7.68
4-Methylpyridine·ICN	-8.32	-8.20	-0.12	1.46
4-Ethylpyridine·ICN	-8.71	-8.24	-0.47	5.70
3,4-Dimethylpyridine·ICN	-9.03	-8.56	-0.47	5.49
4-Dimethylamminopyridine·ICN	-10.30	-10.02	-0.28	2.79
2-Fluoropyridine·Br_2	-6.58	-6.17	-0.41	6.65
2-Chloropyridine·Br_2	-6.91	-6.34	-0.57	8.99
3,5-Dichloropyridine·Br_2	-7.78	-7.15	-0.63	8.81
3-Chloropyridine·Br_2	-9.16	-8.62	-0.54	6.26
3-Acetylpyridine·Br_2	-9.82	-9.25	-0.57	6.16
2-Isopropylpyridine·Br_2	-11.25	-10.47	-0.78	7.45
4-Acetylpyridine·Br_2	-9.62	-9.20	-0.42	4.57
2-Ethylpyridine·Br_2	-10.93	-10.55	-0.38	3.60
Pyridine·Br_2	-10.85	-10.46	-0.39	3.73

2,4,6-Trimethylpyridine·Br_2	-11.25	-11.01	-0.24	2.18
2-Methylpyridine·Br_2	-11.34	-10.76	-0.58	5.39
4-Methylpyridine·Br_2	-11.55	-11.14	-0.41	3.68
4-Ethylpyridine·Br_2	-11.68	-11.21	-0.47	4.19
3,4-Dimethylpyridine·Br_2	-12.27	-11.69	-0.58	4.96
4-Dimethylamminopyridine·Br_2	-13.99	-13.68	-0.31	2.27
2-Fluoropyridine·BrCN	-4.81	-4.61	-0.20	4.34
2-Chloropyridine·BrCN	-4.84	-4.51	-0.33	7.32
3,5-Dichloropyridine·BrCN	-4.55	-4.27	-0.28	6.56
3-Chloropyridine·BrCN	-5.35	-5.09	-0.26	5.11
3-Acetylpyridine·BrCN	-5.40	-5.22	-0.18	3.45
2-Isopropylpyridine·BrCN	-5.29	-5.65	0.36	-6.37
4-Acetylpyridine·BrCN	-5.45	-5.31	-0.14	2.64
2-Ethylpyridine·BrCN	-5.88	-5.69	-0.19	3.34
Pyridine·BrCN	-6.28	-6.05	-0.23	3.80
2,4,6-Trimethylpyridine·BrCN	-5.78	-5.59	-2.22	3.40
2-Methylpyridine·BrCN	-6.07	-5.88	-0.19	3.23
4-Methylpyridine·BrCN	-6.66	-6.41	-0.25	3.90
4-Ethylpyridine·BrCN	-6.54	-6.44	-0.10	1.55
3,4-Dimethylpyridine·BrCN	-7.03	-6.66	-0.37	5.56
4-Dimethylamminopyridine·BrCN	-7.69	-7.68	-0.01	0.13

Table S10 Two-centre IQA interaction energies (a.u.) and exchange-correlation contributions (a.u.) computed at B3LYP, MP2 and M06-2X levels of theory for the set of complexes of substituted pyridines with I_2, ICN, Br_2 and BrCN molecules.

Complex	B3LYP		MP2		M06-2X	
	$E_{IQA}^{Inter(X,N)}$	$VX_{IQA}^{Inter(X,N)}$	$E_{IQA}^{Inter(X,N)}$	$VX_{IQA}^{Inter(X,N)}$	$E_{IQA}^{Inter(X,N)}$	$VX_{IQA}^{Inter(X,N)}$
2-Fluoropyridine·I_2	*-0.086698*	*-0.042407*	*-0.088547*	*-0.040943*	*-0.089778*	*-0.037132*
2-Chloropyridine·I_2	*-0.086089*	*-0.042639*	*-0.087935*	*-0.041374*	*-0.091222*	*-0.038314*
3,5-Dichloropyridine·I_2	*-0.096179*	*-0.050266*	*-0.098089*	*-0.048664*	*-0.096431*	*-0.043034*
3-Chloropyridine·I_2	*-0.107508*	*-0.056165*	*-0.109372*	*-0.054165*	*-0.111032*	*-0.049571*
3-Acetylpyridine·I_2	*-0.111488*	*-0.058494*	*-0.113217*	*-0.056281*	*-0.117376*	*-0.052730*
2-Isopropylpyridine·I_2	*-0.112832*	*-0.058159*	*-0.114052*	*-0.055881*	*-0.123705*	*-0.054102*
4-Acetylpyridine·I_2	*-0.112174*	*-0.058654*	*-0.114358*	*-0.056606*	*-0.116209*	*-0.052023*
2-Ethylpyridine·I_2	*-0.114519*	*-0.059082*	*-0.115764*	*-0.056772*	*-0.126480*	*-0.055578*
Pyridine·I_2	*-0.119890*	*-0.062571*	*-0.121663*	*-0.060207*	*-0.128732*	*-0.057654*
2,4,6-Trimethylpyridine·I_2	*-0.109834*	*-0.056199*	*-0.110548*	*-0.053636*	*-0.122581*	*-0.052698*
2-Methylpyridine·I_2	*-0.117117*	*-0.060704*	*-0.118408*	*-0.058299*	*-0.130165*	*-0.057624*
4-Methylpyridine·I_2	*-0.125060*	*-0.065150*	*-0.126271*	*-0.062597*	*-0.136468*	*-0.061136*
4-Ethylpyridine·I_2	*-0.125604*	*-0.065435*	*-0.126705*	*-0.062875*	*-0.137040*	*-0.061436*
3,4-Dimethylpyridine·I_2	*-0.128739*	*-0.066946*	*-0.130069*	*-0.064461*	*-0.141900*	*-0.063587*
4-Dimethylamminopyridine·I_2	*-0.143044*	*-0.074118*	*-0.143875*	*-0.071275*	*-0.163606*	*-0.073351*
2-Fluoropyridine·ICN	*-0.133324*	*-0.032730*	*-0.134021*	*-0.032075*	*-0.147528*	*-0.032673*
2-Chloropyridine·ICN	*-0.131593*	*-0.032640*	*-0.131932*	*-0.032200*	*-0.146213*	*-0.033041*
3,5-Dichloropyridine·ICN	*-0.138014*	*-0.037712*	*-0.137921*	*-0.036971*	*-0.149280*	*-0.036146*

3-Chloropyridine·ICN	-0.145353	-0.041249	-0.145278	-0.040191	-0.156817	-0.039128
3-Acetylpyridine·ICN	-0.148484	-0.042879	-0.148153	-0.041618	-0.161039	-0.041001
2-Isopropylpyridine·ICN	-0.148042	-0.041557	-0.147381	-0.040277	-0.165601	-0.041388
4-Acetylpyridine·ICN	-0.148119	-0.042740	-0.148531	-0.041625	-0.159809	-0.040511
2-Ethylpyridine·ICN	-0.150070	-0.042505	-0.149388	-0.041178	-0.166726	-0.042087
Pyridine·ICN	-0.154099	-0.045475	-0.153990	-0.044077	-0.166354	-0.043075
2,4,6-Trimethylpyridine·ICN	-0.142763	-0.038275	-0.141762	-0.036855	-0.161866	-0.038990
2-Methylpyridine·ICN	-0.151533	-0.043574	-0.150989	-0.042179	-0.167647	-0.042873
4-Methylpyridine·ICN	-0.157693	-0.047160	-0.157153	-0.045589	-0.170107	-0.044587
4-Ethylpyridine·ICN	-0.158025	-0.047334	-0.157452	-0.045744	-0.171191	-0.045119
3,4-Dimethylpyridine·ICN	-0.160565	-0.048368	-0.160100	-0.046797	-0.173597	-0.045975
4-Dimethylamminopyridine·ICN	-0.169770	-0.053096	-0.169307	-0.051154	-0.183609	-0.050298
2-Fluoropyridine·Br_2	-0.088683	-0.052667	-0.090310	-0.048714	-0.078471	-0.040656
2-Chloropyridine·Br_2	-0.089745	-0.054237	-0.091353	-0.050379	-0.080202	-0.042315
3,5-Dichloropyridine·Br_2	-0.097663	-0.061380	-0.099016	-0.056955	-0.085001	-0.047119
3-Chloropyridine·Br_2	-0.107261	-0.067677	-0.108229	-0.062613	-0.096624	-0.053987
3-Acetylpyridine·Br_2	-0.111065	-0.070416	-0.111747	-0.065042	-0.101698	-0.057283
2-Isopropylpyridine·Br_2	-0.114816	-0.072800	-0.114712	-0.067261	-0.111645	-0.062909
4-Acetylpyridine·Br_2	-0.111777	-0.070640	-0.112807	-0.065436	-0.102178	-0.057476
2-Ethylpyridine·Br_2	-0.116204	-0.073649	-0.116069	-0.068056	-0.113548	-0.064173
Pyridine·Br_2	-0.118016	-0.074751	-0.118366	-0.069061	-0.112286	-0.063623
2,4,6-Trimethylpyridine·Br_2	-0.118595	-0.075385	-0.117327	-0.069440	-0.118060	-0.066705
2-Methylpyridine·Br_2	-0.118388	-0.075110	-0.118145	-0.069354	-0.116827	-0.066302
4-Methylpyridine·Br_2	-0.122436	-0.077550	-0.122001	-0.071554	-0.118380	-0.067323
4-Ethylpyridine·Br_2	-0.123022	-0.077950	-0.122416	-0.071898	-0.119752	-0.068216
3,4-Dimethylpyridine·Br_2	-0.125703	-0.079637	-0.125153	-0.073604	-0.123659	-0.070561
4-Dimethylamminopyridine·Br_2	-0.137222	-0.086974	-0.135387	-0.080240	-0.140333	-0.080625
2-Fluoropyridine·BrCN	-0.089902	-0.025363	-0.093043	-0.024698	-0.096240	-0.025549
2-Chloropyridine·BrCN	-0.089085	-0.025480	-0.092040	-0.024968	-0.095197	-0.025773
3,5-Dichloropyridine·BrCN	-0.092026	-0.028625	-0.094673	-0.027818	-0.095738	-0.027403
3-Chloropyridine·BrCN	-0.097130	-0.031204	-0.099753	-0.030068	-0.099937	-0.029059
3-Acetylpyridine·BrCN	-0.099129	-0.032378	-0.101593	-0.031041	-0.101735	-0.029948
2-Isopropylpyridine·BrCN	-0.099894	-0.031907	-0.102162	-0.030515	-0.104438	-0.030280
4-Acetylpyridine·BrCN	-0.098833	-0.032206	-0.101818	-0.031004	-0.101376	-0.029772
2-Ethylpyridine·BrCN	-0.101237	-0.032622	-0.103538	-0.031191	-0.105763	-0.030856
Pyridine·BrCN	-0.103098	-0.034248	-0.105748	-0.032745	-0.105053	-0.031151
2,4,6-Trimethylpyridine·BrCN	-0.097981	-0.030338	-0.099993	-0.028866	-0.105005	-0.029863
2-Methylpyridine·BrCN	-0.102171	-0.033361	-0.104545	-0.031857	-0.106178	-0.031261
4-Methylpyridine·BrCN	-0.105421	-0.035390	-0.107757	-0.033709	-0.106960	-0.031885
4-Ethylpyridine·BrCN	-0.105621	-0.035502	-0.107968	-0.033801	-0.107021	-0.031910
3,4-Dimethylpyridine·BrCN	-0.107299	-0.036207	-0.109720	-0.034483	-0.108433	-0.032391
4-Dimethylamminopyridine·BrCN	-0.113091	-0.039374	-0.115453	-0.037251	-0.113485	-0.034536

Table S11 Coefficients α, β and γ for equation (9)

Method	Halogenated moiety	α	β	γ

B3LYP	I2	-12.41286	42.35189	0.47152
	ICN	-0.16102	25.25273	0.28341
	Br2	-5.94104	28.73528	0.35666
	BrCN	8.12130	12.26137	0.15997
M06-2X	I2	-4.65949	30.15337	0.34466
	ICN	4.95269	16.81142	0.20515
	Br2	0.41146	19.59114	0.24720
	BrCN	11.51001	6.83501	0.10806
M11	I2	0.72101	24.29193	0.26593
	ICN	9.09386	12.38314	0.15012
	Br2	8.26139	10.77193	0.14022
	BrCN	12.90656	5.63129	0.09146
wB97X	I2	-0.40868	25.16801	0.29005
	ICN	5.63493	16.39659	0.20453
	Br2	3.37602	16.94035	0.21518
	BrCN	7.63549	12.27664	0.16037
WB97XD	I2	-4.87557	31.44823	0.35373
	ICN	4.34074	18.29537	0.22059
	Br2	2.30592	18.03367	0.22979
	BrCN	7.57027	12.30136	0.15953
MP2	I2	-1.08731	22.08328	0.2647
	ICN	5.67649	12.89818	0.16912
	Br2	2.42356	13.75378	0.19533
	BrCN	5.75734	11.31261	0.15136

Table S12 Delocalization indices calculated with equation (9) at different levels of theory for the set of complexes of substituted pyridines with I_2, ICN, Br_2 and BrCN molecules.

Complex	B3LYP	MP2	M06-2X	M11	ωB97X	ωB97XD
2-Fluoropyridine·I_2	*0.241838*	*0.207209*	*0.210408*	*0.190701*	*0.205855*	*0.214276*
2-Chloropyridine·I_2	*0.243789*	*0.209238*	*0.215684*	*0.197561*	*0.209257*	*0.214687*

3,5-Dichloropyridine·I_2	*0.281079*	*0.240618*	*0.240466*	*0.21291*	*0.231243*	*0.24047*
3-Chloropyridine·I_2	*0.311123*	*0.265126*	*0.272425*	*0.233973*	*0.25114*	*0.264061*
3-Acetylpyridine·I_2	*0.323861*	*0.275132*	*0.28888*	*0.245452*	*0.262246*	*0.278441*
2-Isopropylpyridine·I_2	*0.31767*	*0.27037*	*0.290856*	*0.247436*	*0.253339*	*0.270594*
4-Acetylpyridine·I_2	*0.32474*	*0.276928*	*0.28554*	*0.244996*	*0.261133*	*0.276252*
2-Ethylpyridine·I_2	*0.325619*	*0.27535*	*0.298995*	*0.254898*	*0.259273*	*0.276729*
Pyridine·I_2	*0.342704*	*0.29175*	*0.311205*	*0.265376*	*0.279316*	*0.296011*
2,4,6-Trimethylpyridine·I_2	*0.311687*	*0.26179*	*0.285763*	*0.247093*	*0.243047*	*0.258383*
2-Methylpyridine·I_2	*0.329737*	*0.281422*	*0.307237*	*0.264741*	*0.26708*	*0.282962*
4-Methylpyridine·I_2	*0.354168*	*0.300618*	*0.325947*	*0.276261*	*0.2893*	*0.307577*
4-Ethylpyridine·I_2	*0.355834*	*0.301888*	*0.328118*	*0.279585*	*0.290677*	*0.309699*
3,4-Dimethylpyridine·I_2	*0.359999*	*0.30788*	*0.337334*	*0.286974*	*0.296828*	*0.3168*
4-Dimethylamminopyridine·I_2	*0.396456*	*0.338059*	*0.385587*	*0.32745*	*0.330699*	*0.354288*
2-Fluoropyridine·ICN	*0.192117*	*0.16773*	*0.187674*	*0.168324*	*0.192602*	*0.193465*
2-Chloropyridine·ICN	*0.193315*	*0.168691*	*0.189904*	*0.172964*	*0.195056*	*0.194326*
3,5-Dichloropyridine·ICN	*0.220346*	*0.191182*	*0.207739*	*0.188089*	*0.21476*	*0.218292*
3-Chloropyridine·ICN	*0.239267*	*0.206069*	*0.22294*	*0.200322*	*0.227781*	*0.231211*
3-Acetylpyridine·ICN	*0.248372*	*0.212268*	*0.23258*	*0.207136*	*0.234107*	*0.237824*
2-Isopropylpyridine·ICN	*0.23769*	*0.204826*	*0.229533*	*0.206939*	*0.22473*	*0.229926*
4-Acetylpyridine·ICN	*0.247457*	*0.212978*	*0.230465*	*0.205367*	*0.233598*	*0.23704*
2-Ethylpyridine·ICN	*0.243176*	*0.208927*	*0.234029*	*0.210183*	*0.229777*	*0.234125*
Pyridine·ICN	*0.259548*	*0.223286*	*0.24245*	*0.21666*	*0.243288*	*0.246615*
2,4,6-Trimethylpyridine·ICN	*0.221539*	*0.18883*	*0.217785*	*0.199334*	*0.212666*	*0.217366*
2-Methylpyridine·ICN	*0.246837*	*0.213221*	*0.238973*	*0.215852*	*0.235014*	*0.238297*
4-Methylpyridine·ICN	*0.26834*	*0.229453*	*0.249107*	*0.222112*	*0.248919*	*0.252863*
4-Ethylpyridine·ICN	*0.269396*	*0.23087*	*0.252709*	*0.224358*	*0.250297*	*0.254661*
3,4-Dimethylpyridine·ICN	*0.273294*	*0.234168*	*0.255591*	*0.225863*	*0.252502*	*0.257693*
4-Dimethylamminopyridine·ICN	*0.296837*	*0.253713*	*0.277001*	*0.244073*	*0.272204*	*0.279438*
2-Fluoropyridine·Br_2	*0.282519*	*0.234676*	*0.220418*	*0.201624*	*0.209653*	*0.225345*
2-Chloropyridine·Br_2	*0.291872*	*0.242303*	*0.229189*	*0.212201*	*0.217982*	*0.231533*
3,5-Dichloropyridine·Br_2	*0.324089*	*0.270334*	*0.253328*	*0.228857*	*0.240798*	*0.260994*
3-Chloropyridine·Br_2	*0.354404*	*0.293589*	*0.283665*	*0.25194*	*0.260321*	*0.2872*
3-Acetylpyridine·Br_2	*0.367204*	*0.304617*	*0.299822*	*0.264028*	*0.271586*	*0.300676*
2-Isopropylpyridine·Br_2	*0.374157*	*0.310831*	*0.322143*	*0.281248*	*0.274206*	*0.305301*
4-Acetylpyridine·Br_2	*0.369192*	*0.306679*	*0.301483*	*0.264406*	*0.271543*	*0.299862*
2-Ethylpyridine·Br_2	*0.381129*	*0.314989*	*0.328785*	*0.285146*	*0.279667*	*0.309366*
Pyridine·Br_2	*0.387028*	*0.320911*	*0.329437*	*0.285602*	*0.290294*	*0.316411*
2,4,6-Trimethylpyridine·Br_2	*0.38591*	*0.318718*	*0.33881*	*0.293155*	*0.273528*	*0.308792*
2-Methylpyridine·Br_2	*0.382917*	*0.318701*	*0.337083*	*0.292603*	*0.285303*	*0.312419*
4-Methylpyridine·Br_2	*0.397587*	*0.331248*	*0.346113*	*0.298589*	*0.300651*	*0.327581*
4-Ethylpyridine·Br_2	*0.401386*	*0.332528*	*0.349501*	*0.300707*	*0.301097*	*0.328805*
3,4-Dimethylpyridine·Br_2	*0.407352*	*0.338882*	*0.360535*	*0.307489*	*0.306563*	*0.336584*
4-Dimethylamminopyridine·Br_2	*0.442631*	*0.366781*	*0.407374*	*0.351052*	*0.337918*	*0.375004*
2-Fluoropyridine·BrCN	*0.147445*	*0.129552*	*0.145129*	*0.131615*	*0.150001*	*0.148646*
2-Chloropyridine·BrCN	*0.148625*	*0.131227*	*0.146323*	*0.135715*	*0.151042*	*0.148906*
3,5-Dichloropyridine·BrCN	*0.166222*	*0.14524*	*0.15625*	*0.146316*	*0.163725*	*0.165143*
3-Chloropyridine·BrCN	*0.179526*	*0.155826*	*0.164695*	*0.154058*	*0.172667*	*0.175134*
3-Acetylpyridine·BrCN	*0.185137*	*0.160052*	*0.168615*	*0.156899*	*0.176522*	*0.179719*
2-Isopropylpyridine·BrCN	*0.18091*	*0.156229*	*0.16942*	*0.161864*	*0.173001*	*0.176927*

4-Acetylpyridine·BrCN	0.185045	0.160448	0.1687	0.156743	0.176536	0.179228
2-Ethylpyridine·BrCN	0.184457	0.159133	0.17147	0.162406	0.17547	0.179531
Pyridine·BrCN	0.194471	0.167567	0.175248	0.164385	0.182377	0.185779
2,4,6-Trimethylpyridine·BrCN	0.170426	0.148082	0.163926	0.158224	0.166005	0.168318
2-Methylpyridine·BrCN	0.187967	0.16202	0.173966	0.164836	0.178432	0.181839
4-Methylpyridine·BrCN	0.199754	0.171955	0.178549	0.167478	0.186092	0.190121
4-Ethylpyridine·BrCN	0.201037	0.172619	0.179191	0.168435	0.18671	0.190879
3,4-Dimethylpyridine·BrCN	0.203252	0.173977	0.180204	0.16773	0.186601	0.191202
4-Dimethylamminopyridine·BrCN	0.218841	0.187318	0.191071	0.179419	0.198006	0.2029

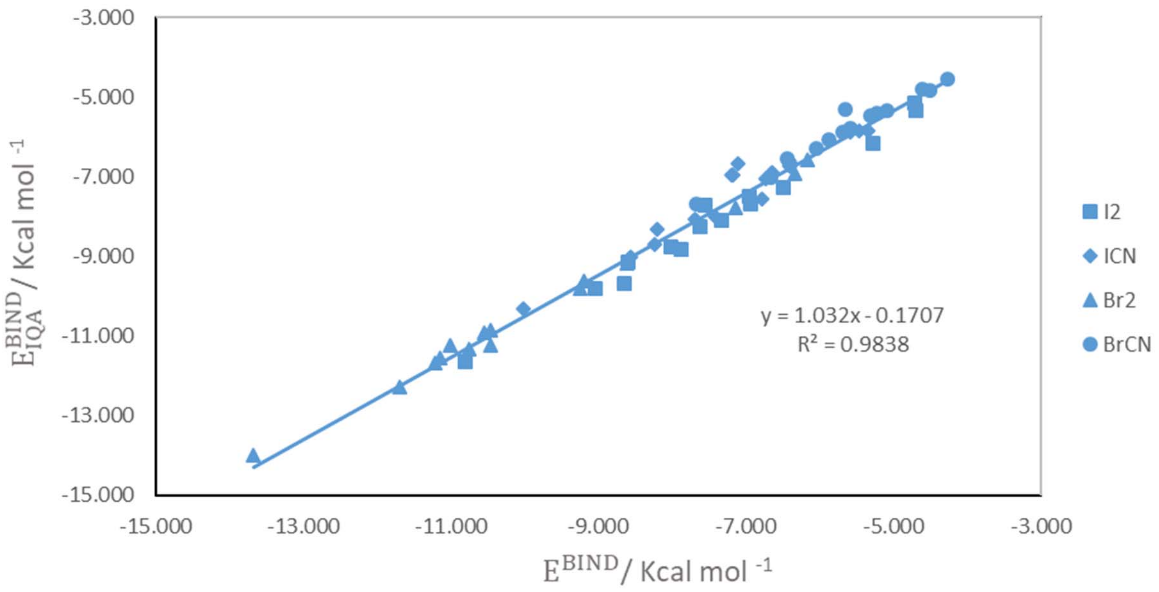

Figure S1 Relationship between IQA binding energy, E_{IQA}^{BIND}, and exact binding energy, E_{BIND}, obtained at B3LYP level of theory.

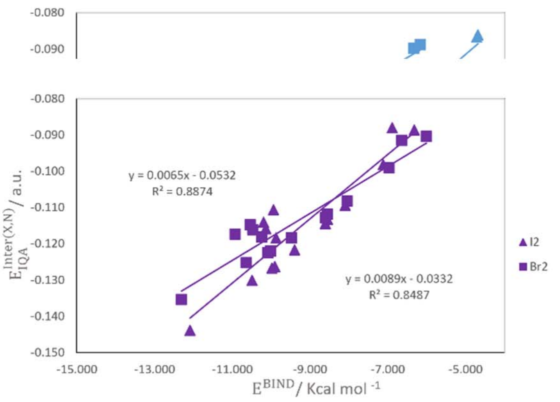

2a

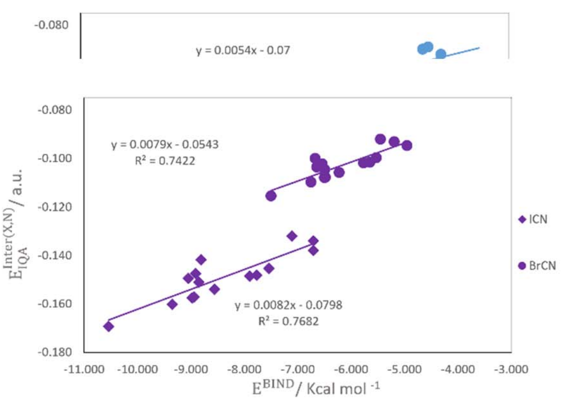

2b

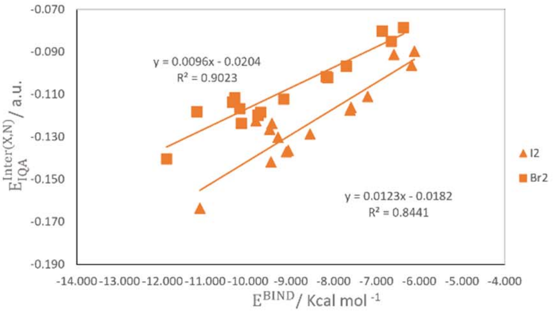

3a

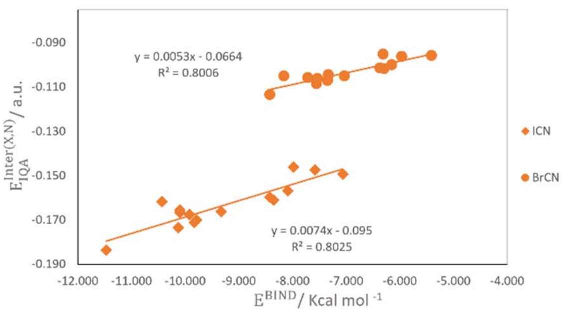

3b

Figure S2 Relationship between IQA halogen bond energy and binding energy obtained for complexes formed with (a) X_2 and (b) XCN molecules at (1) B3LYP, (2) MP2 and (3) M06-2X levels of theory.

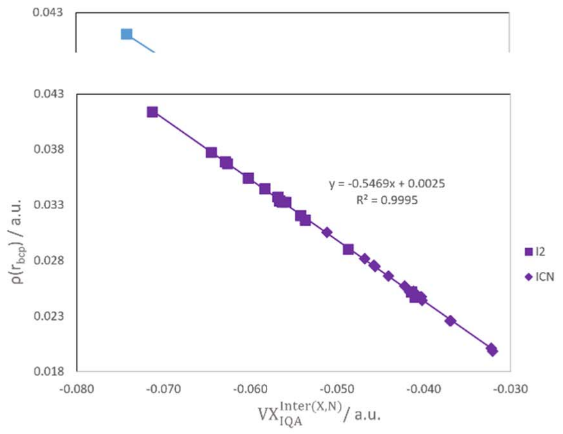

2a

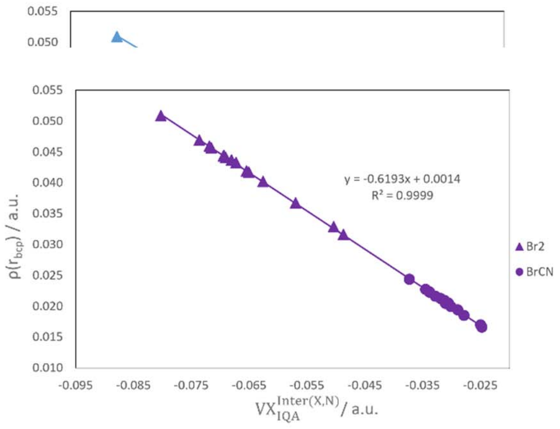

2b

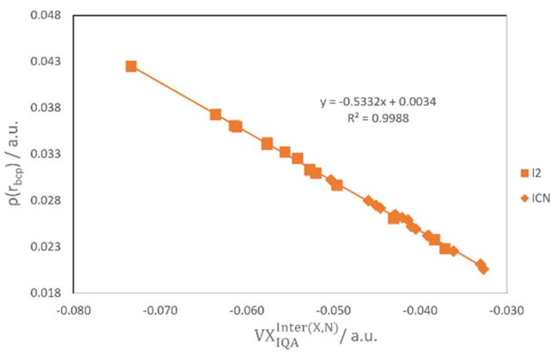

3a

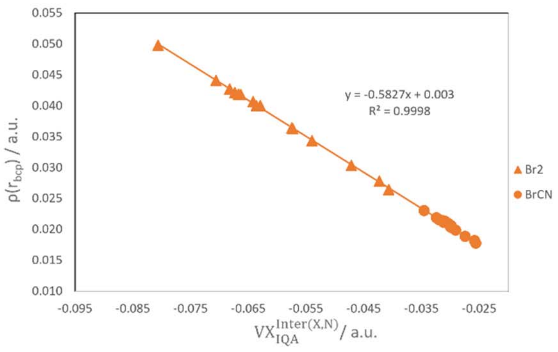

3b

Figure S3 Relationships between electron density at $\mathbf{r}_{bcp}$ and exchange-correlation contribution to IQA halogen bond energy obtained at (1) B3LYP, (2) MP2 and (3) M06-2X levels of theory for complexes formed with molecules containing (a) iodine (b) bromine atoms.

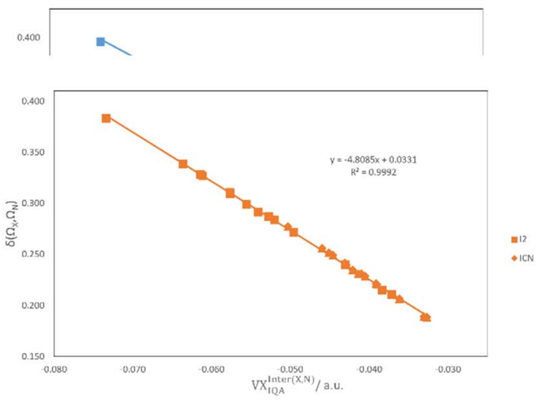

2a

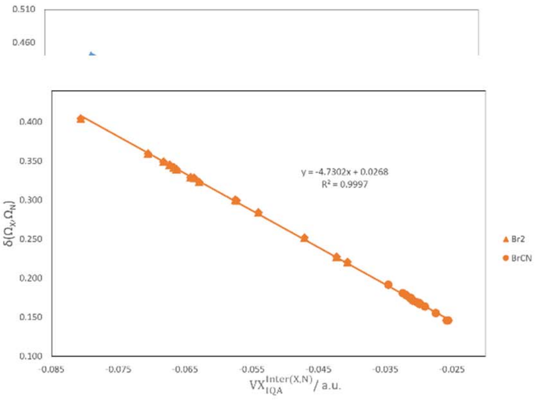

2b

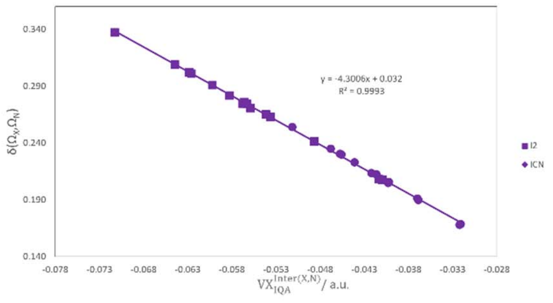

3a

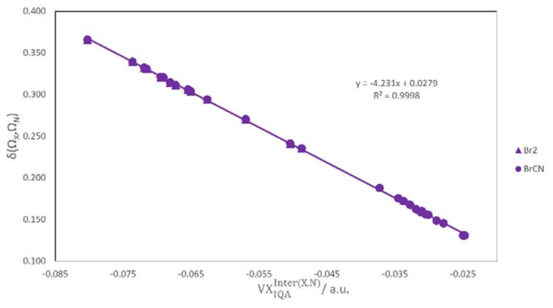

3b

Figure S4 Relationships between delocalization index and exchange-correlation contribution to IQA halogen bond energy obtained at (1) B3LYP, (2) MP2 and (3) M06-2X levels of theory for complexes formed with molecules containing (a) iodine (b) bromine atoms.

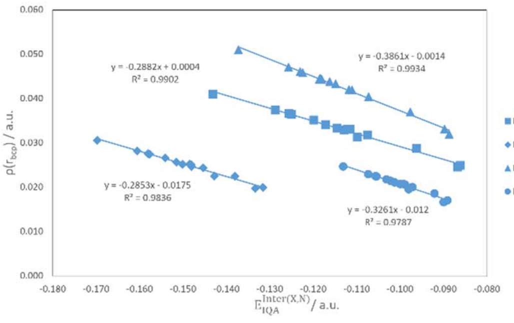

1

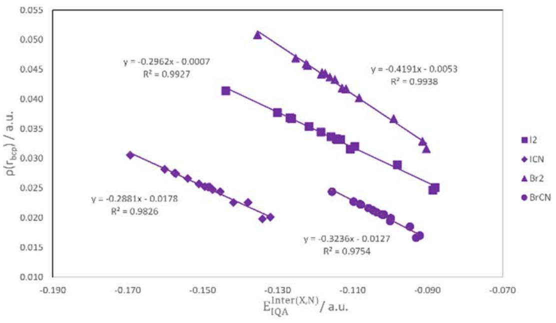

2

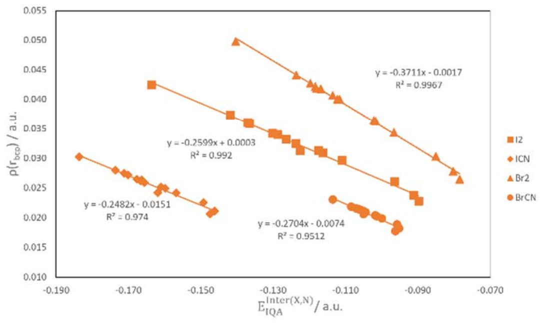

3

Figure S5 Relationship between electron density at r_{bcp} and IQA halogen bond energy obtained at (1) B3LYP, (2) MP2 and (3) M06-2X levels of theory.

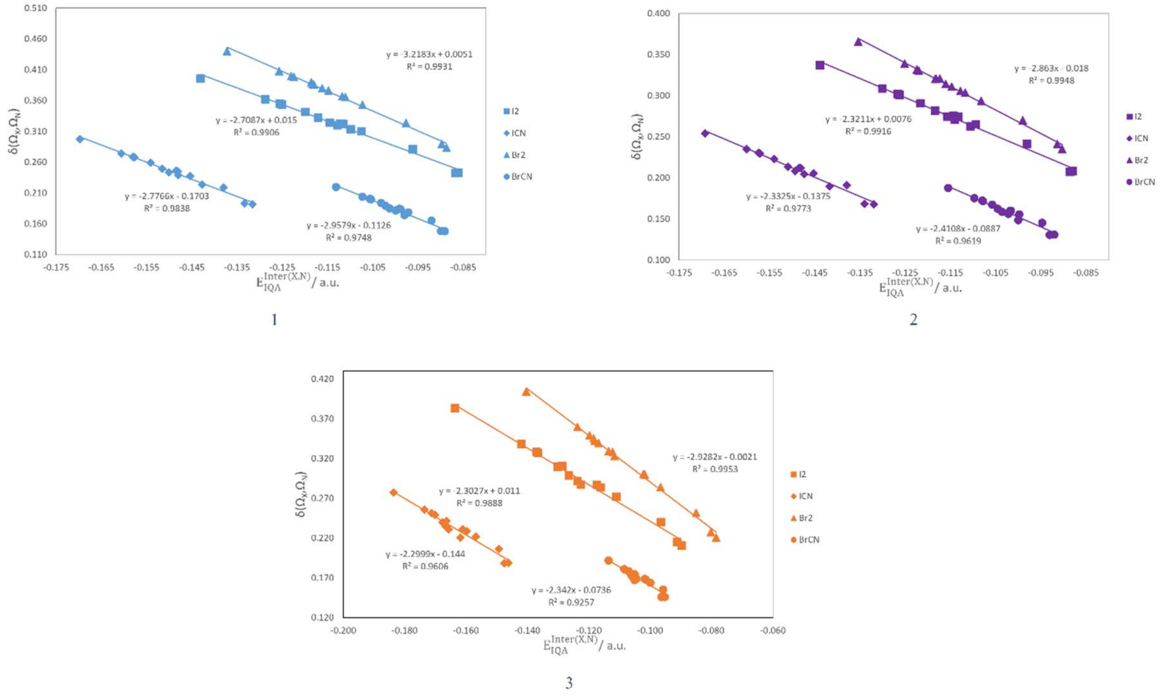

Figure S6 Relationship between delocalization index and IQA halogen bond energy obtained at (1) B3LYP, (2) MP2 and (3) M06-2X levels of theory.

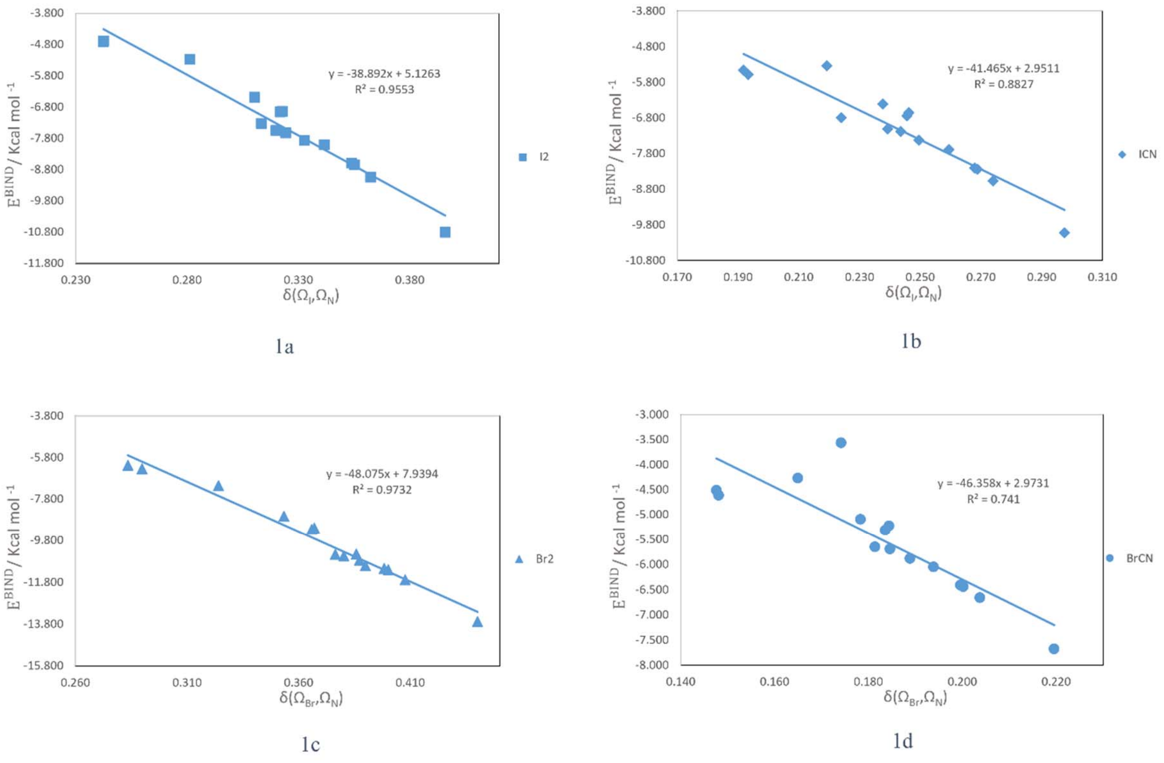

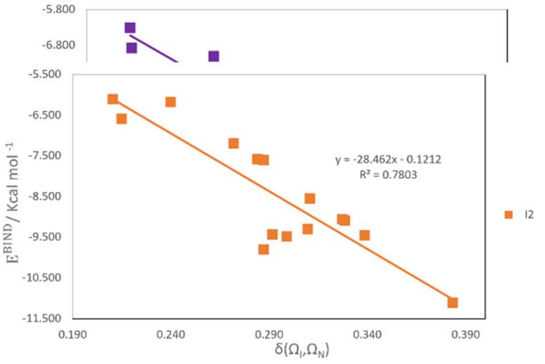
y = -28.462x - 0.1212
R² = 0.7803
I2
E^BIND / Kcal mol -1
δ(Ω_I,Ω_N)

3a

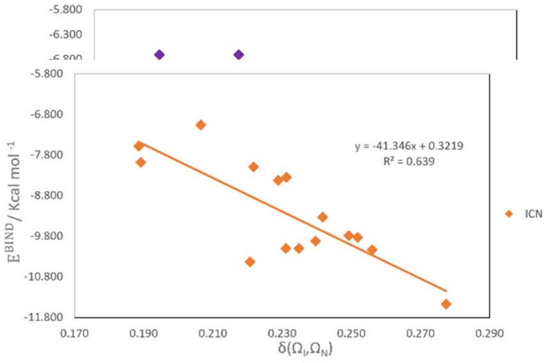
y = -41.346x + 0.3219
R² = 0.639
ICN
E^BIND / Kcal mol -1
δ(Ω_I,Ω_N)

3b

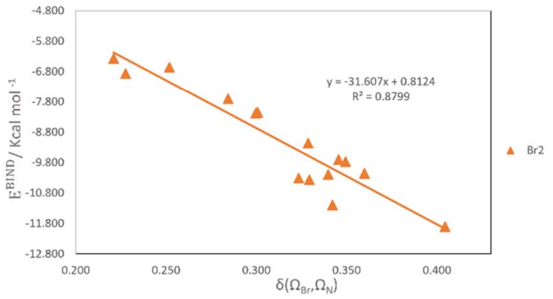
y = -31.607x + 0.8124
R² = 0.8799
Br2
E^BIND / Kcal mol -1
δ(Ω_Br,Ω_N)

3c

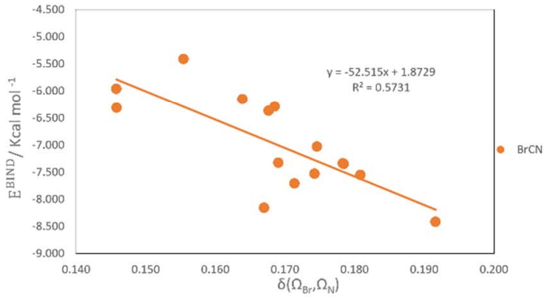
y = -52.515x + 1.8729
R² = 0.5731
BrCN
E^BIND / Kcal mol -1
δ(Ω_Br,Ω_N)

3d

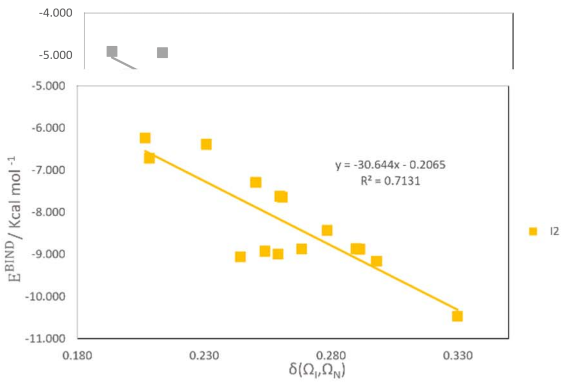

y = -30.644x - 0.2065
R² = 0.7131
I2
E^{BIND} / Kcal mol -1
δ(Ω$_I$,Ω$_N$)
-4.000
-5.000
-5.000
-6.000
-7.000
-8.000
-9.000
-10.000
-11.000
0.180
0.230
0.280
0.330

5a

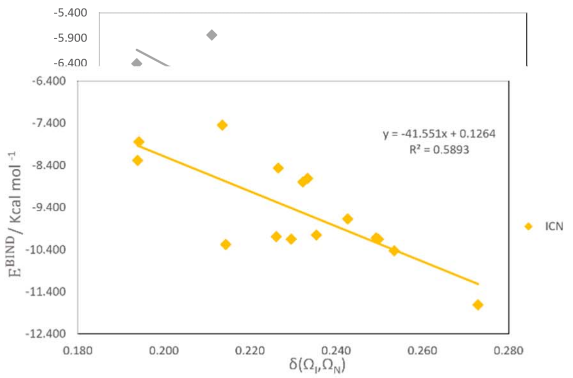

y = -41.551x + 0.1264
R² = 0.5893
ICN
E^{BIND} / Kcal mol -1
δ(Ω$_I$,Ω$_N$)
-5.400
-5.900
-6.400
-6.400
-7.400
-8.400
-9.400
-10.400
-11.400
-12.400
0.180
0.200
0.220
0.240
0.260
0.280

5b

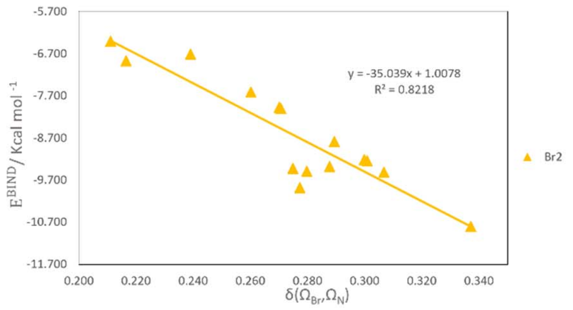

y = -35.039x + 1.0078
R² = 0.8218
Br2
E^{BIND} / Kcal mol -1
δ(Ω$_{Br}$,Ω$_N$)
-5.700
-6.700
-7.700
-8.700
-9.700
-10.700
-11.700
0.200
0.220
0.240
0.260
0.280
0.300
0.320
0.340

5c

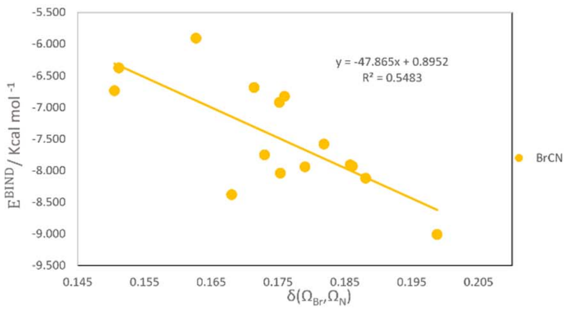

y = -47.865x + 0.8952
R² = 0.5483
BrCN
E^{BIND} / Kcal mol -1
δ(Ω$_{Br}$,Ω$_N$)
-5.500
-6.000
-6.500
-7.000
-7.500
-8.000
-8.500
-9.000
-9.500
0.145
0.155
0.165
0.175
0.185
0.195
0.205

5d

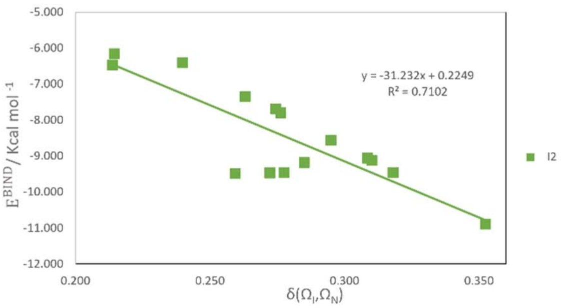

6a

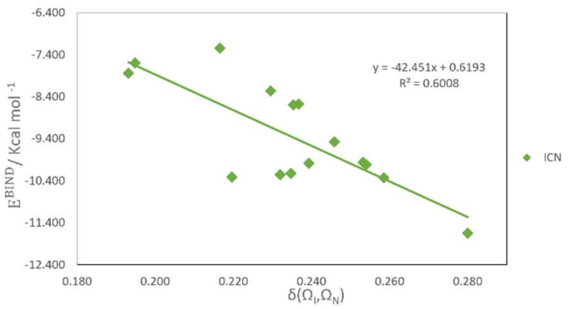

6b

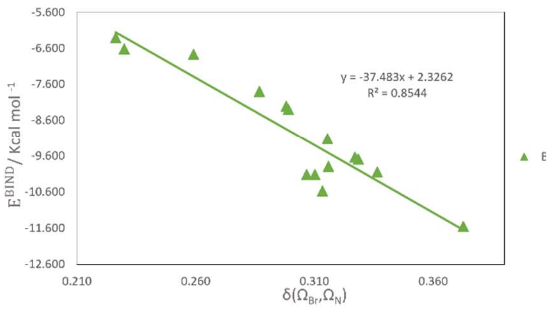

6c

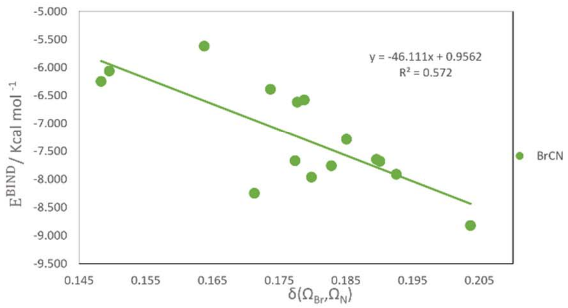

6d

Figure S7 Relationship between binding energy and delocalization index obtained at (1) B3LYP, (2) MP2, (3) M06-2X, (4) M11, (5) ωB97X and (6) ωB97XD levels of theory for complexes formed with (a) I_2, (b) ICN, (c) Br_2 and (d) BrCN.

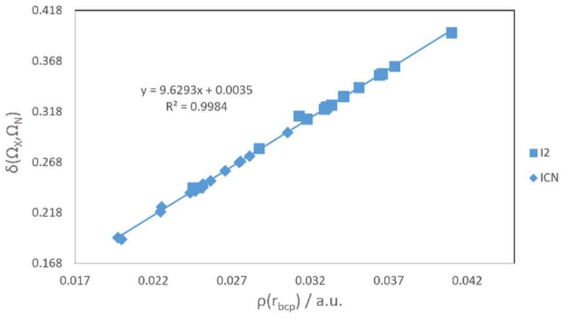

1a

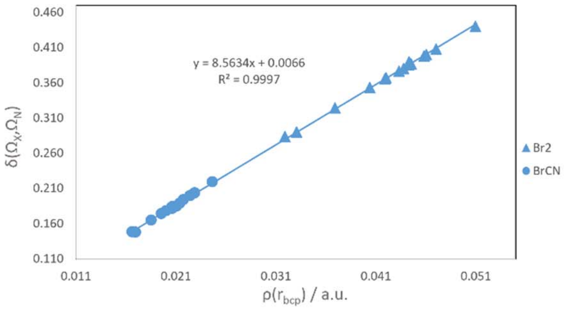

1b

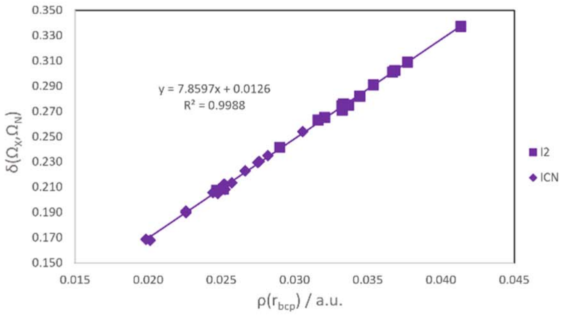

2a

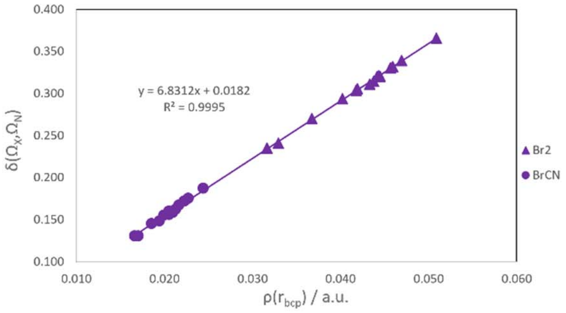

2b

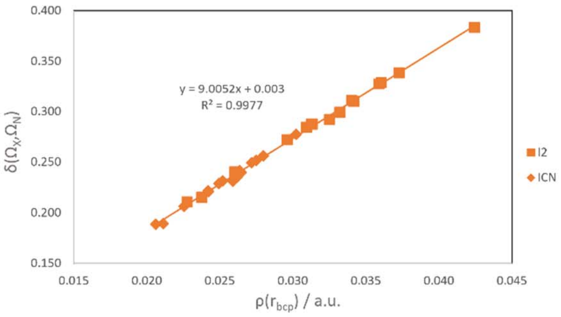

3a

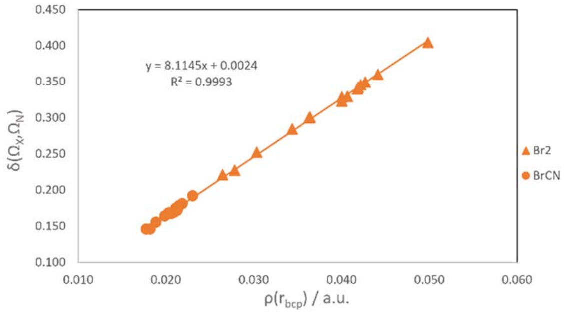

3b

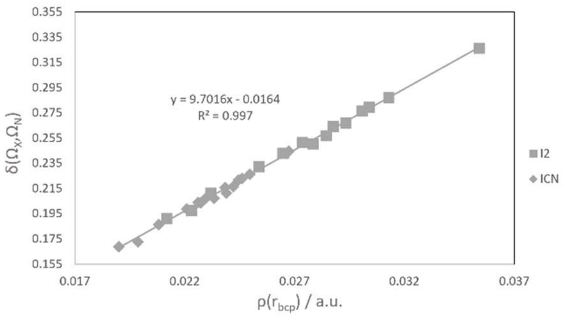

4a

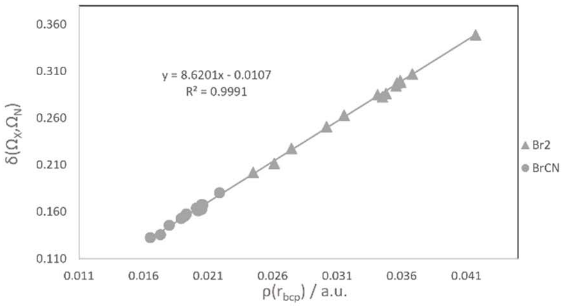

4b

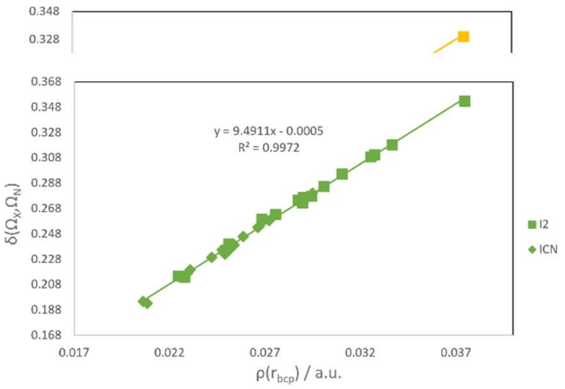

6a

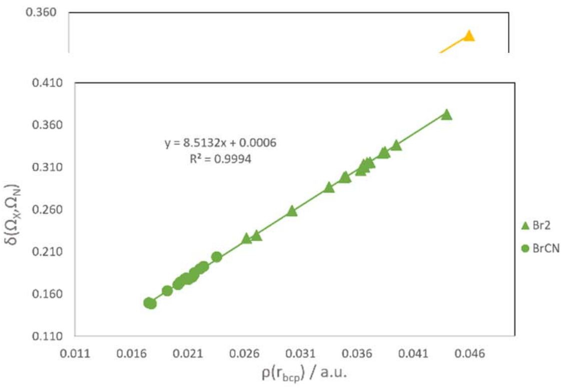

6b

Figure S8 Relationship between delocalization index and electron density at the *X*···N $\mathbf{r}_{bcp}$ obtained at (1) B3LYP, (2) MP2, (3) M06-2X, (4) M11, (5) ωB97X and (6) ωB97XD levels of theory for complexes formed with molecules containing (a) iodine and (b) bromine atoms.
